# Modern contraceptive utilisation and its associated factors among reproductive age women in high fertility regions of Ethiopia: a multilevel analysis of Ethiopia Demographic and Health Survey

**DOI:** 10.1136/bmjopen-2022-066432

**Published:** 2023-02-14

**Authors:** Tadele Biresaw Belachew, Wubshet Debebe Negash, Desalegn Anmut Bitew, Desale Bihonegn Asmamaw

**Affiliations:** 1 Department of Health Systems and Policy, University of Gondar, Gondar, Ethiopia; 2 Department of Reproductive Health, Institute of Public Health, University of Gondar, Gondar, Ethiopia

**Keywords:** Nutrition, Epidemiology, Economics, Health policy, EPIDEMIOLOGY

## Abstract

**Objective:**

This study is aimed to assess the magnitude of modern contraceptives utilisation and associated factors among reproductive age women in high fertility regions of Ethiopia.

**Design:**

Cross-sectional study.

**Setting:**

High fertility regions of Ethiopian.

**Participants:**

A total weighted sample of 3822 married reproductive age women.

**Methods:**

In this study, data were obtained from the recent Ethiopian Demographic and Health Surveys. A total weighted sample of 3822 women of reproductive age was included. A multilevel mixed-effect binary logistic regression model was fitted to identify the significant associated factors of modern contraceptive utilisation. Statistical significance was determined using adjusted OR (AOR) with 95% CI.

**Results:**

The overall modern contraceptive utilisation was 29.75% (95% CI 28.2% to 31.2%). Among the factors associated with utilisation were women’s age 25–34 years (AOR 1.3; 95% CI 1.01 to 1.66) and ≥35 (AOR 1.71; 95% CI 1.37 to 2.70), husband’s occupation (AOR 1.49; 95% CI 1.03 to 1.99), number of alive children: 1–4 (AOR 2.20; 95% CI 1.47 to 3.30), 5–8 (AOR 1.74; 95% CI 1.09 to 2.77), husband’s desired number of children (AOR 0.77; 95% CI 0.61 to 0.96), residency (AOR 2.37; 95% CI 1.20 to 4.67), community media exposure (AOR 1.77; 95% CI 1.01 to 3.08), region (AOR 0.13; 95% CI 0.03 to 0.52) and religion (AOR 0.49; 95% CI 0.36 to 0.66) were significantly associated with modern contraceptive utilisation.

**Conclusion:**

Modern contraceptives utilisation in high fertility regions of Ethiopia was low. Women age, husband occupation, number of living children, husband’s desired number of children, residency, community media exposure, region and religion were significantly associated with modern contraceptive utilisation. Therefore, to improve the utilisation of modern contraceptives, public health policy makers should consider creating awareness through mass media, male involvement in family planning, as well as family planning programmes, should be encouraged in rural areas.

**STRENGTHS AND LIMITATIONS OF THIS STUDY**

- This study used most recent nationally representative data, which were collected validated and standard data collection tools.
- This study employed multilevel analysis (advanced model) that accounts the correlated nature of Ethiopian Demographic and Health Surveys (EDHS) data in the determination of the estimate.
- The cross-sectional nature of the study does not show the cause and effect relationship between the outcome and the independent factors.
- Moreover, due to EDHS were secondary data, essential factors like attitude and knowledge about contraceptives, partner’s perspectives on contraceptives, and fear of side effects were not available in the EDHS.

## Background

Family planning services support people in making decisions about whether to have children by teaching, advocating and offering birth control methods.^1^ It also prevents pregnancy-related health risks in women, infant mortality, sexually transmitted infections and adolescent pregnancy, while slowing population growth.^2 3^ Additionally, it is the best investment in the healthcare and well-being of women, children and communities.^2^ Access to quality, affordable sexual and reproductive health services like contraceptives and information is crucial to ensure the rights and well-being of women and girls.^4^

The types of contraceptive methods used for family planning can be divided into modern contraception and traditional methods that limit or delay childbirth.^5^ Modern contraceptive methods are more effective than traditional methods that include short-acting (injectable, pills, male condom, female condom, emergency contraceptives),^6–8^ long-acting reversible (hormonal implants and intrauterine devices)^9–11^ and permanent (tubal-ligation (female sterilisation) and vasectomy (male sterilisation))^12–14^ methods that exclude traditional methods which helps to limit number of children and prevent maternal and child morbidity and mortality.^15^

In spite of contraceptive value in improving maternal and child health, a large proportion of women do not use contraceptives with remarkable variations across geographical areas and within country. Though, in the past 20 years, the utilisation of modern contraceptives has increased slightly, from 54.4% in 20th to 57.4% in 21th in the world and 23.6% in 2008 to 28.5% in 2015 in Africa, respectively, as reported by the United Nations Population Fund.^16 17^

According to studies conducted in Asia, modern contraceptive use among married women in Nepal and Bangladesh was 36% and 81.27%, respectively.^18 19^ Similarly, studies in Africa show the high levels of modern contraception utilisation in South Africa, Baringo Kenya and Nigeria at 39.9%, 32%, 31% and 92.7%, respectively.^17 20 21^ Moreover, different studies conducted in Ethiopia also shows that the magnitude of modern contraceptive utilisation ranges from 9.1% to 46.9%.^22–25^

Based on studies conducted in developing countries, factors affecting the use of contraceptives included age, education level, parity, religion, knowledge about modern family planning methods and side effects, method approval by partners and employment status.^26–28^ In addition, studies in Ethiopia found that age, residence, education level of the mother, couple discussions, perception of husband approval, discussions with health extension workers, psychological acceptance, desire for more children, monthly income and number of living children were all affected by modern family planning method use.^22 24 29 30^

In Ethiopia, a variety of strategies have been employed to increase the uptake of contraceptive methods over the last decade. Among the steps taken to increase contraceptive use was the implementation of health extension programmes to change attitudes and improve awareness among the community.^31 32^ To remove health system barriers to the use of contraceptives, the health system expanded health centres and health posts and upgraded primary healthcare units.^31^ In spite of these efforts made at the national level, the proportion of women who use contraceptive methods remains low.^33^

We; therefore, aimed to determine what factors are associated with use of modern contraceptives among women of reproductive age groups in high fertility areas of Ethiopia. It is hoped that the results of the study will help policy makers to make interventions that will help reduce maternal mortality and morbidity through speeding up the utilisation of modern contraceptives.

## Methods

### Study design, period and setting

A community-based cross-sectional survey was conducted using secondary data in the 2016 Ethiopian Demographic and Health Surveys (EDHS),^34^ which was conducted by the Central Statistical Agency in collaboration with the Federal Ministry of Health and the Ethiopian Public Health Institute, which was a national representative sample conducted from 18 January 2016 to 27 June 2016. There are nine regional states in Ethiopia (Tigray, Afar, Amhara, Oromia, Benishangul, Gambela, South Nation, Nationalities and Peoples’ Region (SNNPR), Harari and Somali), and two administrative cities (Addis Ababa and Dire-Dawa), 611 districts and 15 000 Kebeles. Afar, Somali and Oromia regions were included in this study. These regions were selected because they are the high fertility rate regions in Ethiopia with fertility rates above 5.0, a value that is higher than the rate of 4.6 in Ethiopia and 2.47 worldwide.^35 36^

### Data sources

**Figure 1:**
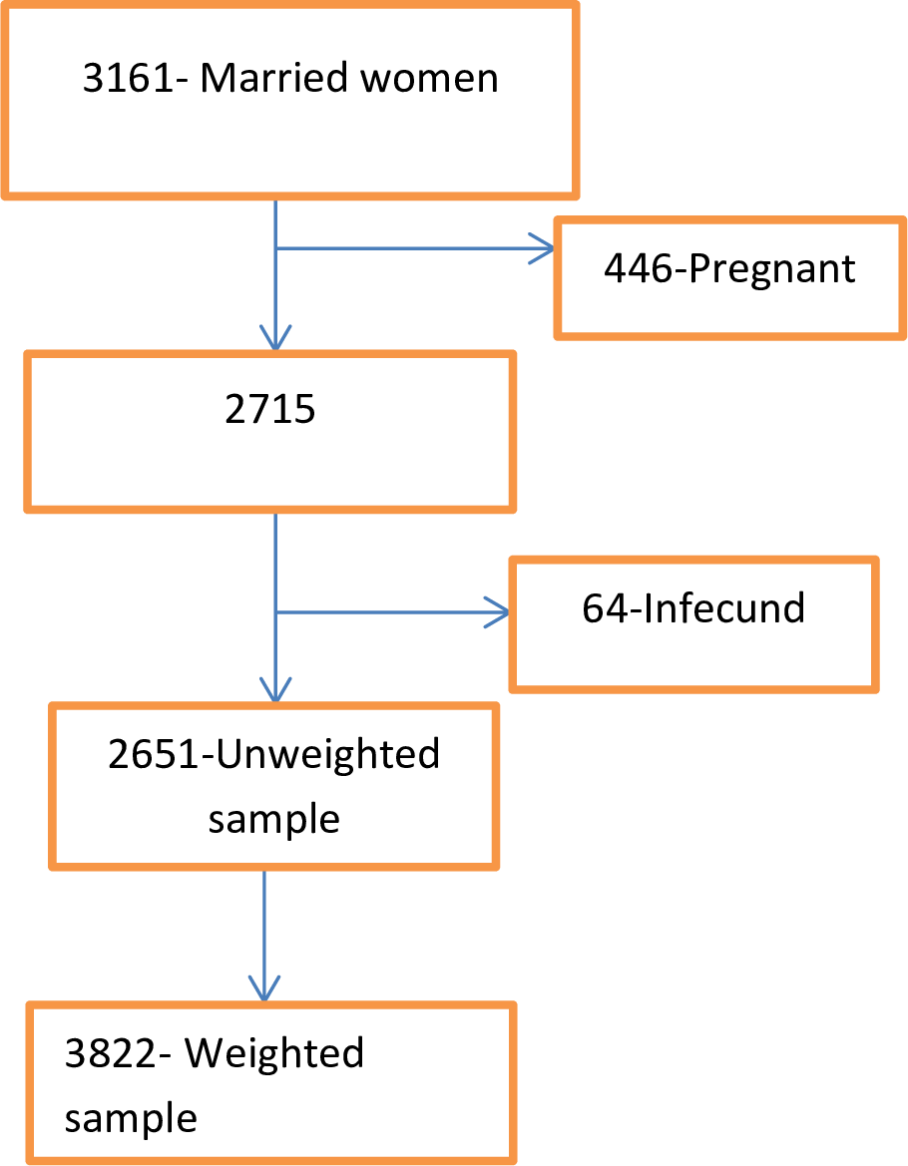
Schematic illustration of women included in high fertility regions of Ethiopia.

The data for these regions were gained from the official database of the Demographic and Health Survey (DHS) programme, www.measuredhs.com after authorisation was granted via online request by explaining the purpose of our study. We extracted dependent and independent variables from the woman record (IR file). DHS is a nationally representative household survey conducted by face-to-face interviews on a wide range of populations. Study participants were selected using a two-stage stratified sampling technique. Enumeration areas (EAs) were randomly selected in the first stage, while households were selected in the second stage.^37^ We calculated the individual weight for women (v005) by multiplying the household weight (hv005) by the inverse of the individual response rate. Before analysis, individual sample weights are generated by dividing (v005) by one million to approximate the number of cases.^10 38 39^ After exclusion of pregnant women and infecund during survey, a total weighted sample of 3822 married reproductive-age women were included from three regions in this study (figure 1).

### Variables and measurements

#### Dependent variable

The outcome variable was modern contraceptive utilisation. In the current study, a woman was considered as modern contraceptive method utiliser if she had been using at least one of the modern contraceptives (female sterilisation, male sterilisation, IUCD, injectable, implants, pills, male condom, female condom and emergency contraception) during EDHS data collection period. Whereas a woman was considered to be non-utiliser of the modern contraceptive method if she had been using traditional methods like rhythm method, lactation amenorrhoea method and withdrawal or if she had not been using any type of contraception during EDHS data collection period.^40^

#### Independent variables

Different independent variables were considered in this study to determine factors associated modern contraceptive utilisation. Community-level variables, residences, region, distance to the health facilities and religion were directly accessed from EDHS data sets. However, community-level poverty, community-level education and community-level media exposure were constructed by aggregating individual-level characteristics at the cluster level.^41^ They were categorised as high or low based on the distribution of the proportion values generated for each community after checking the distribution by using the histogram. The aggregate variable was not normally distributed and the median value was used as a cut-off point for the categorisation (table 1).^41 42^

**Table 1:** List of variables for the assessment of modern contraceptive utilisation

Variables	Description
Age of respondents	15–24, 25–34, 35–49
Educational status of respondents	No formal education, primary education, secondary and above
Husband education	No formal education, primary education, secondary and above
Occupation of respondents	Working, not working
Husband occupation	Working, not working
Wealth index	Poor, middle, rich
No of living children	None, 1–4, 5–8, ≥9
History of abortion	No, Yes
Births in the last 3 years	No birth, one birth, Two and more birth
Visit of health facility in the last 12 months	Yes, no
Husband’s desire for children	Both want the same, husband wants more, husband wants fewer, don’t know
Media exposure	No, yes
Residence	Urban, rural
Community-level poverty	High, low
Community-level media exposure	Low, high
Community-level education	Low, high
Region	Somali, Afar, Oromia
Religion	Orthodox Christian, Muslim, Protestant, others
Distance to the health facilities	Big problem, not big problem

### Data analysis

For data analysis Stata V.16 software was used. To ensure the representativeness of the EDHS sample and obtain reliable estimations and standard errors, data were weighted (v005/1000000) throughout analysis.

Four models fitted: the null model with no explanatory variables, model I with individual factors, model II with community factors, and model III with both individual and community factors. To compare and assess the fitness of nested models, we used the intraclass correlation coefficient (ICC), the median OR and deviation (−2LLR). Model III was the best-fitting model due to its low deviance. In multivariable analysis, variables with a p value less than 0.2 in bivariable analysis were used. Finally, in the multivariable analysis, adjusted ORs (AOR) with 95% CIs and p values less than 0.05 were used to identify factors of modern contraceptive utilisation.

### Patient and public involvement statement

No patient or the public was directly involved in developing the research questions, the design, protocol, data collection tools, results and dissemination plan of the study.

## Results

### Background characteristics of the study participants

A total weighted sample of 3822 individual women participated in this study.

Out of the total respondents, 2489 (65.12 %) women were not attended formal education, 2648 (69.28%) of respondents had no occupation and 2761 (72.24%) of the respondents had no media exposure about family planning. Among the participants, 2071 (54.17%) had 1–4 number of alive children. With regard to their economic status, 1626 (42.55%) women were from the poor wealth quintiles (table 2).

**Table 2:** Individual characteristics of respondents in high fertility regions of Ethiopia (n=3822)

Variables	Categories	Frequency	%
Age of respondents	15–24	891	23.30
	25–34	1689	44.19
	35–49	1242	32.50
Educational status of respondents	No formal education	2489	65.12
	Primary education	1204	31.49
	Secondary and higher	130	3.39
Husband education	No formal education	1795	46.97
	Primary education	1503	39.33
	Secondary and higher	524	13.70
Occupation of respondents	Not working	2648	69.28
	Working	1174	30.72
Husband occupation	Not working	569	14.89
	Working	3253	85.11
Wealth index	Poor	1626	42.55
	Middle	749	19.59
	Rich	1447	37.86
Media exposure	No	2761	72.24
	Yes	1061	27.76
No of living children	None	251	6.56
	1–4	2071	54.17
	5–8	1339	35.02
	≥9	162	4.24
History of abortion	No	216	55.18
	Yes	175	44.82
Births in the last 3 years	No birth	1502	39.28
	One birth	1885	49.31
	Two and more births	436	11.41
Visit of health facility in the last 12 months	No	2187	57.22
	Yes	1635	42.78
Husband’s desire for children	Both want the same	1298	34.03
	Husband wants more	1162	30.45
	Husband wants fewer	239	6.27
	Don’t know	1116	29.26

### Community-level factors

Of the respondents 3390 (88.69%) were rural dwellers. Nearly 60% respondents were Muslims. Among the respondents 3485 (91.18%) were from Oromia region. About 3072 (80.38 %) of the respondents were from communities with low proportion of poverty level. Almost two-thirds (63.24%) of women had media exposure and 2235 (58.48%) of participant’s perceived as big problem to access health facilities (table 3).

**Table 3:** Community-level characteristics of respondents in high fertility regions of Ethiopia (n=3822)

Variables	Categories	Frequency	%
Residence	Rural	3390	88.69
	Urban	432	11.31
Community-level poverty	High	750	19.62
	Low	3072	80.38
Community media exposure	Low	1405	36.76
	High	2417	63.24
Community-level education	Low	1214	31.77
	High	2608	68.23
Region	Afar	82	2.15
	Oromo	3485	91.18
	Somali	255	6.67
Religion	Orthodox Christian	756	19.77
	Protestant	693	18.13
	Muslim	2240	58.61
	Others	133	3.48
Distance to health facilities	Big problem	2235	58.48
	Not big problem	1587	41.52

### Modern contraceptive utilisation

**Figure 2:**
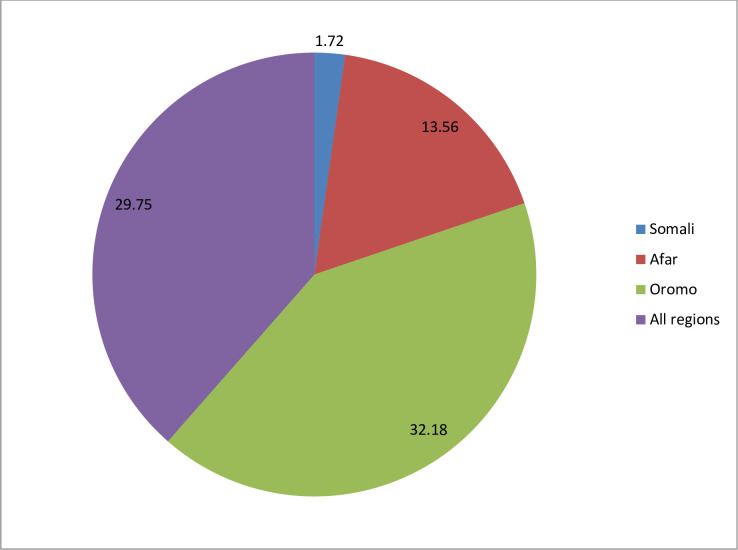
Magnitude of modern contraceptive utilisation in high fertility regions of Ethiopia.

Overall, the magnitude of modern contraceptive utilisation in high fertility regions of Ethiopia was 29.75% (95% CI 28.2% to 31.2%), with Oromia recording the highest magnitude of 32.18% (figure 2).

### Multilevel analysis of factors

In terms of the individual-level factors, the study showed that reproductive age women aged 25–34 and 35–49 were more likely to use modern contraceptive (AOR 1.3; 95% CI 1.01 to 1.66), (AOR 1.71; 95% CI 1.37 to 2.70) than compared with those aged 15–24 women, respectively. Women who had a husband with occupation during the time of survey had higher odds (AOR 1.49; 95% CI 1.03 to 1.99) to use modern contraceptive compared with who did not work. Women who had 1–4 number of living children were 2.2 times modern contraceptive use (AOR 2.20; 95% CI 1.47 to 3.30) than who had no children and reproductive age women who had 5–8 living children were more likely to use modern contraceptive (AOR 1.74; 95% CI 1.09 to 2.77) compared with women who had no living children. Women who had a partner whose desire for children to have more were 23% less likely to use modern contraceptive (AOR 0.77; 95% CI 0.61 to 0.96) compared with both want the same to have children and also women who had a Partner whose desire for children unknown were 29% less likely to use modern contraceptive (AOR 0.71; 95% CI 0.56 to 0.89) than both want the same to have children.

Regarding community-level factors, the odds of modern contraceptive utilisation were high among reproductive age women who had community media exposure (AOR 1.77; 95% CI 1.01 to 3.08) compared with their counterparts. Women who were residing in urban area were 2.37 times contraceptive use than living in rural area (AOR 2.37; 95% CI 1.20 to 4.67). In addition, women in Somali region were 87% less likely (AOR 0.13; 95% CI 0.03 to 0.52) to use modern contraceptive compared with women in Oromia region. Moreover, women who follow Muslim in religion were 51% less likely to use modern contraceptive (AOR 0.49; 95% CI 0.36 to 0.66) than Orthodox Christian followers (table 4).

**Table 4:** Multivariable analyses for factors affecting modern contraceptive utilisation (n=3822)

Variables	Model 0	Model 1 AOR (95% CI)	Model 2 AOR (95% CI)	Model 3 AOR (95% CI)
Individual-level characteristics				
Age of respondents				
15–24		1		1
25–34		1.39 (1.08 to 1.77)		1.29 (1.01 to 1.66)*
35–49		0.58 (0.43 to 0.79)		1.71 (1.37 to 2.70)*
Educational status of the respondents				
No formal education		1		1
Primary education		1.15 (0.93 to 1.44)		1.06 (0.85 to 1.33)
Secondary and higher		1.33 (0.78 to 2.30)		1.02 (.59 to 1.79)
Husband education				
No formal education		1		1
Primary education		1.14 (0.93 to 1.5)		1.05 (0.85 to 1.29)
Secondary and higher		1.07 (0.76 to 1.47)		0.96 (0.68 to 1.34)
Husband occupation				
Not working		1		1
Working		1.55 (1.16 to 2.06)		1.49 (1.03 to 1.99)*
Wealth index				
Poor		1		1
Middle		1.24 (0.96 to 1.59)		1.02 (0.79 to 1.31)
Rich		1.53 (1.19 to 1.98)		1.19 (0.92 to 1.54)
Media exposure				
No		1		1
Yes		1.24 (0.99 to 1.54)		1.15 (0.92 to 1.43)
No of living children				
None		1		1
1–4		2.18 (1.46 to 3.25)		2.20 (1.47 to 3.30)*
5–8		1.70 (1.08 to 2.69)		1.74 (1.09 to 2.77)*
≥9		1.10 (0.51 to 2.39)		1.21 (0.55 to 2.65)
Visit of health facility in the last 12 months				
No		1		1
Yes		1.32 (1.10 to 1.58)		1.29 (0.74 to 1.54)
Partner’s desire for children				
Both want the same		1		
Husband wants more		0.75 (0.61 to 0.94)		0.77 (0.61 to 0.96)*
Husband wants fewer		0.84 (0.58 to 1.22)		0.82 (0.56 to 1.18)
Don’t know		0.72 (0.57 to 0.90)		0.71 (0.56 to 1.89)
Community-level variables				
Residency				
Rural			1	1
Urban			2.86 (1.48 to 5.55)	2.37 (1.20 to 4.67)*
Community-level media exposure				
Low			1	1
High			1.92 (1.09 to 3.39)	1.77 (1.01 to 3.08)*
Community-level poverty				
High			0.32 (0.15 to 0.69	0.33 (0.15 to 1.70)
Low			1	**1**
Community-level education				
Low			1	1
High			0.81 (0.45 to 1.43)	0.75 (0.43 to 1.31)
Region				
Oromo			1	1
Afar			2.25 (0.87 to 5.82)	2.19 (0.84 to 5.7)
Somali			0.11 (0.03 to 0.44)	0.13 (0.033 to 0.52)*
Religion				
Orthodox Christian			1	1
Protestant			1.11 (0.83 to 1.50)	1.08 (0.79 to 1.47)
Muslim			0.52 (0.38 to 0.71)	0.49 (0.36 to 0.66)*
Others			0.61 (0.33 to 1.15)	0.61 (0.32 to 1.16)
Random effect results				
Variance (%)	43.7	32.9	17.8	17.2
ICC (%)	41.1	33.3	20.6	19.5
MOR	17.1	14.8	10.9	10.7
PCV	Ref	24.7	59.3	60.6
Model comparison				
Deviance(−2LLR)	3893	3747	3735	3551

*p<0.05.

AOR, adjusted OR; ICC, intraclass correlation coefficient; MOR, median OR; PCV, proportional change in variance.

### Measures of variation

According to the result in the null model, the ICC was 41.1% of the variations of modern contraceptive utilisation among study subjects were attributed by difference at the cluster level, but the rest 58.9% were attributed to individual women factors. The final model indicates that the proportional change in variance value of 0.606 indicates that both individual-level and community-level factors were responsible for about 60.6% of the variation in modern contraceptive utilisation between study subjects. When comparing models/fitness, deviance was used, this model was the best-fitting model, having the lowest deviance (3551) (table 4).

## Discussion

The aim of this study was to investigate the magnitude and associated factors of modern contraceptive utilisation among reproductive age women in high fertility regions of Ethiopia. Slightly above one-fourth 29.75% (95% CI 28.2% to 31.2%) of participants were currently using modern contraceptive. This study is in line with study conducted in Dembia District.^43^ The finding was lower than that reported from 2016 EDHS 35%,^44^ study done in Adama Town 47% and Ethiopia 51.6%.^45 46^ However, this finding is higher than the previous studies conducted in Bale,^22^Gumuz,^47^ Rural women in Ethiopia.^48^ The difference might be due to the different design and sample size.

In this study, the usage of contraceptives was strongly related with women of reproductive age, specifically those age 25–34 and >35 were more likely to use contraceptives than those age 15–24. The findings were similar to those obtained by Nepal.^27^ This may be because younger women (15–24 years) perceive pregnancy risks are low.

The use of contraception by a wife is determined by her husband, and the wife is more likely to use contraception if her husband approves.^49^ In this study, women have a higher likelihood of using contraception if their husband holds a professional, technical or managerial position. This husband’s occupation may reflect husband’s educational status and contributed for contraceptive use by women as indicated.^50^ There is a higher possibility that these women can reside in urban regions.

The use of contraceptive methods was independently associated with the number of children. In comparison to a woman who had no children, women who had 1–4 children and women who had 5–8 children were nearly 2.2 and nearly 2 times more likely, respectively, to have used a modern form of contraception. Additionally, prior studies have shown that using modern contraceptives is more likely when there are a lot of living children.^34 51–55^ This might be because women without children might need to have children in order to have the ideal number of children.^56^

The current study also showed that the husband’s desire to have more children was inversely related to modern contraceptive use. Women with husbands who want many children were 23% less likely to use modern contraceptives than women who want the same number of children with their husbands. This is congruent with the results of secondary data analyses of the DHS of Bangladesh, Burkina Faso and Mali, which showed that a husband’s desire to have children influenced a woman’s use of modern contraception when she was of reproductive age.^57 58^

In regard to community-level factors, according to this study, residence was independently associated with modern contraceptive use. Urban women of reproductive age are about three times more likely than rural women to use contemporary contraceptives. This finding is in line with secondary data analysis of Indian, Afghan, Nigerian and Bangladeshi DHS, which showed that urban resident women were more likely than rural resident women to use contemporary contraceptives.^48 59–61^ This could be caused by a variety of factors. Urban women tend to be more educated, earn more money, have better access to health facilities and have better media access than rural women, all of which lead to higher modern contraceptive utilisation rates. Women in rural areas also require more children to help them with field work, which negatively impacts their use of modern contraceptives.^40 62–66^

Women who were exposed to the community media had a higher likelihood of using modern methods of contraception than women who were not. The finding is in line with the results.^34 48 52 54 67^ This is due to the possibility that exposure to mass media could play a significant role in raising awareness and inspiring women to take contemporary contraceptives.

In addition woman living in Somali region of Ethiopia was 87% less likely to use modern contraceptive compared with women in Oromia region. The reason could be different access to health information and different availability of maternal health services like family planning.^68^

Moreover, women who were Muslim religious followers were less likely to use modern contraceptive than Orthodox Christian religious followers. It is possible that the low rate of family planning methods, such as the usage of contemporary contraceptives, among Muslims and Protestants in Ethiopia is the cause of this.^69^ In addition, there is evidence that Muslims are less likely to approve of the use of contraceptives and have a negative attitude about family planning.^70^

### Strengths and limitations

This study used most recent nationally representative data, which were collected with validated and standardised data collection tools. This also employed multilevel analysis (advanced model) that accounts the correlated nature of EDHS data in the determination of the estimate. Despite the above advantages, the cross sectional nature of the study does not show the cause and effect relationship between the outcome and the independent factors. Moreover, due to EDHS were secondary data, essential factors like attitude and knowledge about contraceptives, partner’s perspectives on contraceptives, and fear of side effects were not available in the EDHS.

## Conclusion

Modern contraceptives utilisation in high fertility regions of Ethiopia was low. Women age, husband occupation, number of living children, husband’s desired number of children, residency, community media exposure, region and religion were significantly associated with modern contraceptive utilisation. Therefore, to improve the utilisation of modern contraceptives, public health policy makers should consider creating awareness through mass media, male involvement in family planning, as well as family planning programmes, should be encouraged in rural areas.

## Supplementary Material

Reviewer comments

Author's manuscript

## Data Availability

Data are available on reasonable request. Data can be obtained on reasonable request. The data used in this study will be made available to the corresponding author on reasonable request.

## References

[1] Cleland J, Bernstein S, Ezeh A, et al. Family planning: the unfinished agenda. Lancet 2006;368:1810–27. 10.1016/S0140-6736(06)69480-4 17113431

[2] Kesetebirhan A. National guideline for family planning services in Ethiopia. Federal Democratic Republic of Ethiopia, Ministry of Health, 2011.

[3] Nalwadda G, Namutebi M, Volgsten H. Health care providers’ perceptions of family planning and contraception education for adolescents in Kampala, Uganda-a qualitative study. Sex Reprod Healthc 2019;21:15–20. 10.1016/j.srhc.2019.05.001 31395228

[4] Organization WH. Family planning: a global handbook for providers. World Health Organization, 2018.

[5] Ajong AB, Njotang PN, Kenfack B, et al. Contraceptive method mix and preference: a focus on long acting reversible contraception in urban Cameroon. PLoS One 2018;13:e0202967. 10.1371/journal.pone.0202967 30138474 PMC6107242

[6] Ugaz JI, Chatterji M, Gribble JN, et al. Regional trends in the use of short-acting and long-acting contraception accessed through the private and public sectors. Int J Gynaecol Obstet 2015;130 Suppl 3:E3–7. 10.1016/j.ijgo.2015.03.021 26001703

[7] Tibaijuka L, Odongo R, Welikhe E, et al. Factors influencing use of long-acting versus short-acting contraceptive methods among reproductive-age women in a resource-limited setting. BMC Womens Health 2017;17:25. 10.1186/s12905-017-0382-2 28376779 PMC5379613

[8] Hopkins K, Hubert C, Coleman-Minahan K, et al. Unmet demand for short-acting hormonal and long-acting reversible contraception among community college students in Texas. J Am Coll Health 2018;66:360–8. 10.1080/07448481.2018.1431901 29405858 PMC6692077

[9] Espey E, Ogburn T. Long-acting reversible contraceptives: intrauterine devices and the contraceptive implant. Obstet Gynecol 2011;117:705–19. 10.1097/AOG.0b013e31820ce2f0 21343774

[10] Negash WD, Belachew TB, Asmamaw DB. Long acting reversible contraceptive utilization and its associated factors among modern contraceptive users in high fertility sub-saharan Africa countries: a multi-level analysis of recent demographic and health surveys. Arch Public Health 2022;80:224. 10.1186/s13690-022-00977-1 36280847 PMC9590189

[11] Bahamondes L, Fernandes A, Monteiro I, et al. Long-acting reversible contraceptive (larcs) methods. Best Pract Res Clin Obstet Gynaecol 2020;66:28–40. 10.1016/j.bpobgyn.2019.12.002 32014434

[12] Alemayehu M, Belachew T, Tilahun T. Factors associated with utilization of long acting and permanent contraceptive methods among married women of reproductive age in mekelle town, tigray region, north Ethiopia. BMC Pregnancy Childbirth 2012;12:1–9. 10.1186/1471-2393-12-6 22280163 PMC3297532

[13] Getahun DS, Wolde HF, Muchie KF, et al. Utilization and determinants of long term and permanent contraceptive methods among married reproductive age women at janamora district, Northwest Ethiopia. BMC Res Notes 2018;11:836. 10.1186/s13104-018-3942-0 30477564 PMC6257970

[14] Zenebe CB, Adefris M, Yenit MK, et al. Factors associated with utilization of long-acting and permanent contraceptive methods among women who have decided not to have more children in Gondar city. BMC Womens Health 2017;17:75. 10.1186/s12905-017-0432-9 28877687 PMC5588745

[15] Creanga AA, Gillespie D, Karklins S, et al. Low use of contraception among poor women in Africa: an equity issue. Bull World Health Organ 2011;89:258–66. 10.2471/BLT.10.083329 21479090 PMC3066524

[16] Miller K, Miller RA, Askew I, et al. Clinic-based family planning and reproductive health services in Africa: findings from situation analysis studies. 1998.

[17] Innocent A, Sunday Y, A MM. Knowledge, attitude and uptake of family planning services among women of reproductive age group attending outpatient clinic at a tertiary health institution in edo state, Nigeria. J Public Health Epidemiol 2019;11:63–70. 10.5897/JPHE2018.1112

[18] Sharma SK, Ghimire DR, Pratap N. Ethnic differentials of the impact of family planning program on contraceptive use in Nepal. DemRes 2011;25:837–68. 10.4054/DemRes.2011.25.27

[19] Kibria GMA, Hossen S, Barsha RAA, et al. Factors affecting contraceptive use among married women of reproductive age in Bangladesh. J Mol Stud Med Res 2016;2:70–9. 10.18801/jmsmr.020116.09

[20] Peer N, Morojele N, London L. Factors associated with contraceptive use in a rural area in Western Cape province. S Afr Med J 2013;103:406–12. 10.7196/samj.6201 23725962

[21] Malalu PK. Determinants of use of modern family planning methods: a case of baringo North district, Kenya. SJPH 2014;2:424. 10.11648/j.sjph.20140205.18

[22] Belda SS, Haile MT, Melku AT, et al. Modern contraceptive utilization and associated factors among married pastoralist women in bale eco-region, bale zone, South East Ethiopia. BMC Health Serv Res 2017;17:194. 10.1186/s12913-017-2115-5 28288616 PMC5348813

[23] Mekonnen W, Worku A. Determinants of low family planning use and high unmet need in Butajira district, south central Ethiopia. Reprod Health 2011;8:1–8. 10.1186/1742-4755-8-37 22151888 PMC3248357

[24] Lakew Y, Reda AA, Tamene H, et al. Geographical variation and factors influencing modern contraceptive use among married women in Ethiopia: evidence from a national population based survey. Reprod Health 2013;10:1–10. 10.1186/1742-4755-10-52 24067083 PMC3850415

[25] Tilahun T, Coene G, Luchters S, et al. Family planning knowledge, attitude and practice among married couples in jimma zone, Ethiopia. PLoS One 2013;8:e61335. 10.1371/journal.pone.0061335 23637815 PMC3634055

[26] Adebowale SA, Palamuleni ME. Determinants of unmet need for modern contraception and reasons for non-use among married women in rural areas of Burkina Faso. APS 2014;28:499. 10.11564/28-1-503

[27] Fotso JC, Izugbara C, Saliku T, et al. Unintended pregnancy and subsequent use of modern contraceptive among slum and non-slum women in Nairobi, Kenya. BMC Pregnancy Childbirth 2014;14:1–10. 10.1186/1471-2393-14-224 25012817 PMC4096734

[28] Apanga PA, Adam MA. Factors influencing the uptake of family planning services in the talensi district, Ghana. Pan Afr Med J 2015;20:10. 10.11604/pamj.2015.20.10.5301 25995807 PMC4430143

[29] Medhanyie AA, Desta A, Alemayehu M, et al. Factors associated with contraceptive use in tigray, North Ethiopia. Reprod Health 2017;14:27. 10.1186/s12978-017-0281-x 28228141 PMC5322676

[30] Mohammed A, Woldeyohannes D, Feleke A, et al. Determinants of modern contraceptive utilization among married women of reproductive age group in north shoa zone, Amhara region, Ethiopia. Reprod Health 2014;11:1–7. 10.1186/1742-4755-11-13 24490810 PMC3918182

[31] Kahsay ZH, Hiluf MK, Shamie R, et al. Pregnant women’s intentions to deliver at a health facility in the pastoralist communities of afar, Ethiopia: an application of the health belief model. Int J Environ Res Public Health 2019;16:888. 10.3390/ijerph16050888 30862098 PMC6427120

[32] Sedlander E, Bingenheimer JB, Edberg MC, et al. Understanding modern contraception uptake in one Ethiopian community: a case study. Reprod Health 2018;15:111. 10.1186/s12978-018-0550-3 29925395 PMC6011588

[33] Fantay Gebru K, Mekonnen Haileselassie W, Haftom Temesgen A, et al. Determinants of stunting among under-five children in Ethiopia: a multilevel mixed-effects analysis of 2016 Ethiopian demographic and health survey data. BMC Pediatr 2019;19:176. 10.1186/s12887-019-1545-0 31153381 PMC6544992

[34] Abate MG, Tareke AA. Individual and community level associates of contraceptive use in Ethiopia: a multilevel mixed effects analysis. Arch Public Health 2019;77:46. 10.1186/s13690-019-0371-z 31687139 PMC6820945

[35] African countries with the highest fertility rate | Statista. 2021. Available: https://worldpopulationreview.com/countries/total-fertility-rate

[36] Central statistical agency (CSA)[ethiopia] and ICF. Ethiopia demographic and health survey, addis ababa. Ethiopia and Calverton, Maryland, USA, 2016.

[37] Corsi DJ, Neuman M, Finlay JE, et al. Demographic and health surveys: a profile. Int J Epidemiol 2012;41:1602–13. 10.1093/ije/dys184 23148108

[38] Croft T, Marshall A, Allen C. Guide to DHS statistics. Rockville: ICF, 2018.

[39] Eshete T, Kumera G, Bazezew Y, et al. Determinants of inadequate minimum dietary diversity among children aged 6–23 months in Ethiopia: secondary data analysis from Ethiopian demographic and health survey 2016. Agric & Food Secur 2018;7:1–8. 10.1186/s40066-018-0219-8

[40] Gebre MN, Edossa ZK. Modern contraceptive utilization and associated factors among reproductive-age women in Ethiopia: evidence from 2016 Ethiopia demographic and health survey. BMC Womens Health 2020;20:61. 10.1186/s12905-020-00923-9 32216823 PMC7098091

[41] Liyew AM, Teshale AB. Individual and community level factors associated with anemia among lactating mothers in Ethiopia using data from Ethiopian demographic and health survey, 2016; a multilevel analysis. BMC Public Health 2020;20:775. 10.1186/s12889-020-08934-9 32448212 PMC7247135

[42] Getaneh T, Negesse A, Dessie G, et al. Predictors of unmet need for family planning in Ethiopia 2019: a systematic review and meta analysis. Arch Public Health 2020;78:102. 10.1186/s13690-020-00483-2 33088503 PMC7566059

[43] Debebe S, Andualem Limenih M, Biadgo B. Modern contraceptive methods utilization and associated factors among reproductive aged women in rural dembia district, Northwest Ethiopia: community based cross-sectional study. Int J Reprod Biomed 2017;15:367–74. 29202123 PMC5605858

[44] Ethiopia demographic and health survey 2011. Maryland, USA: Central Statistical Agency Addis Ababa Ethiopia ICF International Calverton, 2012.

[45] Biruk T, Assefa H, Georges R. The prevalence of covert use of contraceptives in nazareth/adama town. Eur J Contracept Reprod Health Care 2008;13:63.

[46] Abraha MW, Nigatu TH. Modeling trends of health and health related indicators in Ethiopia (1995-2008): a time-series study. Health Res Policy Syst 2009;7:29. 10.1186/1478-4505-7-29 20003381 PMC2797494

[47] Adane AA, Bekele YA, Melese E, et al. Modern contraceptive utilization and associated factors among married gumuz women in metekel zone North West Ethiopia. Biomed Res Int 2020;2020:8010327. 10.1155/2020/8010327 32775442 PMC7396020

[48] Fenta SM, Gebremichael SG. Predictors of modern contraceptive usage among sexually active rural women in Ethiopia: a multi-level analysis. Arch Public Health 2021;79:93. 10.1186/s13690-021-00621-4 34088347 PMC8176723

[49] Kamal N. The influence of husbands on contraceptive use by Bangladeshi women. Health Policy Plan 2000;15:43–51. 10.1093/heapol/15.1.43 10731234

[50] Gubhaju B. The influence of wives’ and husbands’ education levels on contraceptive method choice in Nepal, 1996-2006. IPSRH 2009;35:176–85. 10.1363/3517609 20123651

[51] Idris H. Factors affecting the use of contraceptive in Indonesia: analysis from the National socioeconomic survey (susenas). KEMAS 2019;15:117–23. 10.15294/kemas.v15i1.14098

[52] Rutaremwa G, Kabagenyi A, Wandera SO, et al. Predictors of modern contraceptive use during the postpartum period among women in Uganda: a population-based cross sectional study. BMC Public Health 2015;15:262. 10.1186/s12889-015-1611-y 25885372 PMC4372233

[53] Musa A, Assefa N, Weldegebreal F, et al. Factor associated with experience of modern contraceptive use before pregnancy among women who gave birth in kersa HDSS, Ethiopia. BMC Public Health 2016;16:614. 10.1186/s12889-016-3292-6 27443834 PMC4957397

[54] Nsanya MK, Atchison CJ, Bottomley C, et al. Modern contraceptive use among sexually active women aged 15-19 years in north-western Tanzania: results from the adolescent 360 (A360) baseline survey. BMJ Open 2019;9:e030485. 10.1136/bmjopen-2019-030485 PMC6720144 31467055

[55] Devita VD, Rosliza A, Suriani I. Prevalence of modern contraceptive use among reproductive women with hypertension and diabetes in a government hospital in BATAM, Indonesia and its socio-demographic determinants. IJPHCS 2018;5:297–278.

[56] Matovu JKB, Makumbi F, Wanyenze RK, et al. Determinants of fertility desire among married or cohabiting individuals in Rakai, Uganda: a cross-sectional study. Reprod Health 2017;14:2. 10.1186/s12978-016-0272-3 28069056 PMC5223449

[57] O’Regan A, Thompson G. Indicators of young women’s modern contraceptive use in Burkina Faso and Mali from demographic and health survey data. Contracept Reprod Med 2017;2:26. 10.1186/s40834-017-0053-6 29201431 PMC5683538

[58] Hossain MB, Khan MHR, Ababneh F, et al. Identifying factors influencing contraceptive use in Bangladesh: evidence from BDHS 2014 data. BMC Public Health 2018;18:192. 10.1186/s12889-018-5098-1 29378546 PMC5789662

[59] Ezeh OK, Agho KE, Dibley MJ, et al. The impact of water and sanitation on childhood mortality in Nigeria: evidence from demographic and health surveys, 2003-2013. Int J Environ Res Public Health 2014;11:9256–72. 10.3390/ijerph110909256 25198687 PMC4199018

[60] Haq I, Sakib S, Talukder A. Sociodemographic factors on contraceptive use among ever-married women of reproductive age: evidence from three demographic and health surveys in Bangladesh. Med Sci (Basel) 2017;5:31. 10.3390/medsci5040031 29211008 PMC5753660

[61] Dey AK. Socio-demographic determinants and modern family planning usage pattern-an analysis of national family health survey-IV data. Int J Community Med Public Health 2019;6:738. 10.18203/2394-6040.ijcmph20190200

[62] Tekelab T, Melka AS, Wirtu D. Predictors of modern contraceptive methods use among married women of reproductive age groups in Western Ethiopia: a community based cross-sectional study. BMC Womens Health 2015;15:52. 10.1186/s12905-015-0208-z 26183090 PMC4504461

[63] Johnson OE. Determinants of modern contraceptive uptake among Nigerian women: evidence from the National demographic and health survey. Afr J Reprod Health 2017;21:89–95. 10.29063/ajrh2017/v21i3.8 29624932

[64] Osmani AK, Reyer JA, Osmani AR, et al. Factors influencing contraceptive use among women in Afghanistan: secondary analysis of Afghanistan health survey 2012. Nagoya J Med Sci 2015;77:551–61. 26663934 PMC4664587

[65] Adebowale AS, Gbadebo B, Afolabi FR. Wealth index, empowerment and modern contraceptive use among married women in Nigeria: are they interrelated? J Public Health 2016;24:415–26. 10.1007/s10389-016-0738-3

[66] Adebowale SA, Adedini SA, Ibisomi LD, et al. Differential effect of wealth quintile on modern contraceptive use and fertility: evidence from Malawian women. BMC Womens Health 2014;14:40. 10.1186/1472-6874-14-40 24602452 PMC3973841

[67] Agbadi P, Tagoe E, Akosua AF, et al. A multilevel analysis of predictors of modern contraceptive use among reproductive age women in sierra leone: insight from demographic and health surveys. SocArXiv [Preprint]. 10.31235/osf.io/vu5ag

[68] Ababa A. Ethiopia [Google Scholar]. 2013. Available: https://wfpha.confex.com/wfpha/2012/webprogram.Paper10587.html

[69] Roudi-Fahimi F. Islam and family planning: population reference. Washington, DC Bureau; 2004.

[70] Duze MC, Mohammed IZ. Male knowledge, attitudes, and family planning practices in northern Nigeria. Afr J Reprod Health 2006;10:53–65. 10.2307/30032471 17518131

