# Supplementary material for: Modern contraceptive utilisation and its associated factors among reproductive age women in high fertility regions of Ethiopia: a multilevel analysis of Ethiopia Demographic and Health Survey
Source: BMJ Open. 2023 Feb 14;13(2):e066432. doi: 10.1136/bmjopen-2022-066432 (PMC9930559; doi:10.1136/bmjopen-2022-066432)
Supplement: Author's manuscript [file bmjopen-2022-066432.draft_revisions.pdf]

BMJ Open is committed to open peer review. As part of this commitment we make the peer review history of every article we publish publicly available.

When an article is published we post the peer reviewers' comments and the authors' responses online. We also post the versions of the paper that were used during peer review. These are the versions that the peer review comments apply to.

The versions of the paper that follow are the versions that were submitted during the peer review process. They are not the versions of record or the final published versions. They should not be cited or distributed as the published version of this manuscript.

BMJ Open is an open access journal and the full, final, typeset and author-corrected version of record of the manuscript is available on our site with no access controls, subscription charges or pay-per-view fees (http://bmjopen.bmj.com).

If you have any questions on BMJ Open's open peer review process please email info.bmjopen@bmj.com

BMJ Open

Modern contraceptive utilization and its associated factors among reproductive age women in high fertility regions of Ethiopia: A multilevel analysis of Ethiopia Demographic and Health Survey

Journal:	*BMJ Open*
Manuscript ID	bmjopen-2022-066432
Article Type:	Original research
Date Submitted by the Author:	09-Jul-2022
Complete List of Authors:	Belachew, Tadele; University of Gondar, Department of Health Systems and Policy<br>Negash, Wubshet Debebe; University of Gondar, Department of Health Systems and Policy<br>Bitew, Desalegn Anmut; University of Gondar, Department of Reproductive Health, Institute of Public Health<br>Asmamaw, Desale; University of Gondar, Department of Reproductive Health, Institute of Public Health
Keywords:	Nutrition < TROPICAL MEDICINE, Epidemiology < TROPICAL MEDICINE, Economics < TROPICAL MEDICINE

SCHOLARONE™
Manuscripts

I, the Submitting Author has the right to grant and does grant on behalf of all authors of the Work (as defined in the below author licence), an exclusive licence and/or a non-exclusive licence for contributions from authors who are: i) UK Crown employees; ii) where BMJ has agreed a CC-BY licence shall apply, and/or iii) in accordance with the terms applicable for US Federal Government officers or employees acting as part of their official duties; on a worldwide, perpetual, irrevocable, royalty-free basis to BMJ Publishing Group Ltd ("BMJ") its licensees and where the relevant Journal is co-owned by BMJ to the co-owners of the Journal, to publish the Work in this journal and any other BMJ products and to exploit all rights, as set out in our licence.

The Submitting Author accepts and understands that any supply made under these terms is made by BMJ to the Submitting Author unless you are acting as an employee on behalf of your employer or a postgraduate student of an affiliated institution which is paying any applicable article publishing charge ("APC") for Open Access articles. Where the Submitting Author wishes to make the Work available on an Open Access basis (and intends to pay the relevant APC), the terms of reuse of such Open Access shall be governed by a Creative Commons licence – details of these licences and which Creative Commons licence will apply to this Work are set out in our licence referred to above.

Other than as permitted in any relevant BMJ Author's Self Archiving Policies, I confirm this Work has not been accepted for publication elsewhere, is not being considered for publication elsewhere and does not duplicate material already published. I confirm all authors consent to publication of this Work and authorise the granting of this licence.

For peer review only

Modern contraceptive utilization and its associated factors among reproductive age women in high fertility regions of Ethiopia: A multilevel analysis of Ethiopia Demographic and Health Survey

Tadele Biresaw Belachew[1*], Wubshet Debebe Negash[1], Desalegn Anmut Bitew[2], Desale Bihonegn Asmamaw[2]

[1]Department of Health Systems and Policy, Institute of Public Health, College of Medicine and Health Sciences, University of Gondar, Gondar, Ethiopia

[2]Department of Reproductive Health, Institute of Public Health, College of Medicine and Health Sciences, University of Gondar, Gondar, Ethiopia

Authors' addresses

TBB: tadelebiresaw01@gmail.com

WDN: wubshetdn@gmail.com

DAB: Desalegn1227@gmail.com

DBA: desalebihonegn1988@gmail.com

Corresponding author:

Tadele Biresaw Belachew (TBB)

Email: tadelebiresaw01@gmail.com

Phone: +251-918747633

P.O.Box: 196; University of Gondar, Gondar, Ethiopia

For peer review only

For peer review only - http://bmjopen.bmj.com/site/about/guidelines.xhtml

Abstract

Objective: This study is aimed to assess the magnitude of modern contraceptives utilization and associated factors among reproductive age women in high fertility regions of Ethiopia

Design: Cross-sectional study

Setting: High fertility regions of Ethiopian

Participants: A total weighted sample of 3,822 married reproductive age women

Methods: In this study, data were obtained from the recent Demographic and Health Surveys (EDHS). A total weighted sample of 3,822 women of reproductive age was included. A multilevel mixed-effect binary logistic regression model was fitted to identify the significant associated factors of modern contraceptive utilization. Statistical significance was determined using Adjusted Odds Ratio (AOR) with 95% confidence interval.

Results: The overall modern contraceptive utilization was 29.75% (95% CI: 28.2, 31.2). Among the factors associated with utilization were women's age 25-34 years (AOR = 1.3; 95% CI: 1.01, 1.66), and ≥35 (AOR = 1.71; 95% CI: 1.37, 2.70), husband's occupation (AOR = 1.49; 95% CI 1.03, 1.99), number of alive children: 1-4 (AOR = 2.20; 95% CI: 1.47, 3.30), 5-8 (AOR = 1.74; 95% CI:1.09, 2.77), husband's desired number of children (AOR = 0.77; 95% CI: 0.61, 0 .96), residency (AOR = 2.37; 95% CI: 1.20, 4.67), community media exposure (AOR = 1.77; 95% CI: 1.01, 3.08), region (AOR =0.13; 95% CI: 0.03, 0.52), and religion (AOR =0.49; 95% CI: 0.36, 0.66) were significantly associated with modern contraceptive utilization.

Conclusion: Modern contraceptives utilization in high fertility regions of Ethiopia was low. Women age, husband occupation, number of living children, husband's desired number of children, residency, community media exposure, region, and religion were significantly

For peer review only

associated with modern contraceptive utilization. Therefore, to improve the utilization of modern contraceptives, public health policymakers should consider both individual and community level factors when designing family planning programs emphasis should be given to high risk women.

Keywords: Modern contraceptive, multilevel, factors, Ethiopia

Strengths and limitation of study

- This study used most recent nationally representative data, which were collected validated and standard data collection tools.
- This study employed multilevel analysis (advanced model) that accounts the correlated nature of EDHS data in the determination of the estimate.
- The cross sectional nature of the study does not show the cause and effect relationship between the outcome and the independent factors.
- Moreover, due to EDHS were secondary data, essential factors like attitude and knowledge about contraceptives, partner's perspectives on contraceptives, and fear of side effects were not available in the EDHS

For peer review only - http://bmjopen.bmj.com/site/about/guidelines.xhtml

Background

Family Planning services support people in making decisions about whether to have children by teaching, advocating, and offering birth control methods.[1] It also prevents pregnancy-related health risks in women, infant mortality, STIs, and adolescent pregnancy, while slowing population growth.[2,3] Additionally, it is the best investment in the health care and wellbeing of women, children, and communities.[2] Access to quality, affordable sexual and reproductive health services like contraceptives and information is crucial to ensure the rights and wellbeing of women and girls.[4]

The types of contraceptive methods used for family planning can be divided into modern contraception and traditional methods that limit or delay childbirth.[5] Modern contraceptive methods include short-acting, long-acting, and permanent methods that exclude traditional methods which helps to limit number of children and prevent maternal and child morbidity and mortality.[6]

In spite of contraceptive value in improving maternal and child health, a large proportion of women do not use contraceptives with remarkable variations across geographical areas and within country. Though, in the past twenty years, the utilization of modern contraceptives has increased slightly, from 54.4% in 20th to 57.4% in 21th in the world and 23.6% in 2008 to 28.5% in 2015 in Africa respectively, as reported by the United Nations Population Fund (UNFPA).[7,8]

According to studies conducted in Asia, modern contraceptive use among married women in Nepal and Bangladesh was 36% and 81.27%, respectively.[9,10] similarly, studies in Africa show the high levels of modern contraception utilization in South Africa, Baringo Kenya, and Nigeria

For peer review only - http://bmjopen.bmj.com/site/about/guidelines.xhtml

at 39.9%, 32%, 31%, and 92.7%, respectively.[8,11,12] Moreover, different studies conducted in Ethiopia also shows that the magnitude of modern contraceptive utilization ranges from 9.1%-46.9%.[13-16]

Based on studies conducted in developing countries, factors affecting the use of contraceptives included age, education level, parity, religion, knowledge about modern family planning methods and side effects, method approval by partners, and employment status.[17-19] In addition, studies in Ethiopia found that age, residence, education level of the mother, couple discussions, perception of husband approval, discussions with health extension workers, psychological acceptance, desire for more children, monthly income, and number of living children were all affected by modern family planning method use.[13,15,20,21]

In Ethiopia, a variety of strategies have been employed to increase the uptake of contraceptive methods over the last decade. Among the steps taken to increase contraceptive use was the implementation of health extension programs to change attitudes and improve awareness among the community.[22,23] To remove health system barriers to the use of contraceptives, the health system expanded health centers and health posts and upgraded primary health care units.[22] In spite of these efforts made at the national level, the proportion of women who use contraceptive methods remains low.[24]

We therefore aimed to determine what factors are associated with use of modern contraceptives among women of reproductive age groups in high fertility areas of Ethiopia. It is hoped that the results of the study will help policy makers to make interventions that will help reduce maternal mortality and morbidity through speeding up the utilization of modern contraceptives.

For peer review only - http://bmjopen.bmj.com/site/about/guidelines.xhtml

Methods

Study design, period, and setting

A community-based cross-sectional survey was conducted using secondary data in the 2016 EDHS. [25]

The study was a cross-sectional analysis of data from recent EDHS, which was conducted by the Central Statistical Agency (CSA) in collaboration with the Federal Ministry of Health (FMoH) and the Ethiopian Public Health Institute (EPHI), which was a national representative sample conducted from January 18 to June 27, 2016. There are nine regional states in Ethiopia (Tigray, Afar, Amhara, Oromia, Benishangul, Gambela, South Nation, Nationalities and Peoples' Region (SNNPR), Harari, and Somali), and two administrative cities (Addis Ababa and Dire-Dawa), 611 Districts, and 15,000 Kebeles. Afar, Somali and Oromia regions were included in this study. These regions were selected because they are the high fertility rate regions in Ethiopia with fertility rates above 5.0, a value that is higher than the rate of 4.6 in Ethiopia and 2.47 worldwide.[26,27]

Data sources

The data for these regions were gained from the official database of the DHS program, www.measuredhs.com after authorization was granted via online request by explaining the purpose of our study. We extracted dependent and independent variables from the woman record (IR file). DHS is a nationally representative household survey conducted by face-to-face interviews on a wide range of populations. Study participants were selected using a two-stage stratified sampling technique. Enumeration Areas (EAs) were randomly selected in the first stage, while households were selected in the second stage .[28] From married 3,161 women

For peer review only - http://bmjopen.bmj.com/site/about/guidelines.xhtml

participated in the survey. Some exclusion criteria were used to reach the final sample for this study. Pregnant women were 446 during the survey period and were excluded in the first step. The other criteria excluded were 46 not fecund women which were during the survey. After screening for sexual activity and fecundity we reached the final unweight sample size of 2,651 (Fig.1). Finally a total of weighted sample of 3,822 married reproductive age eligible women were included (Table 1).

Table 1. Description of Surveys and sample size characteristics in high fertility regions of Ethiopia (n= 3822)

Regions	Survey year	Weighted sample (n)	Weighted percentage (%)
Afar	2016	82	2.15
Oromo	2016	3,485	91.18
Somali	2016	255	6.67

Variables and measurements

Dependent variable

The outcome variable was modern contraceptive utilization. In the current study a woman was considered as modern contraceptive method utilizer if she had been using at least one of the modern contraceptives (female sterilization, male sterilization, IUCD, injectable, implants, pills, male condom, female condom, emergency contraception, and standard days method) during EDHS data collection period. Whereas a woman was considered to be non-utilizer of the modern contraceptive method if she had been using traditional methods like rhythm method, lactation amenorrhea method, and withdrawal or if she had not been using any type of contraception during EDHS data collection period.[29]

Independent variables

For peer review only

For peer review only - http://bmjopen.bmj.com/site/about/guidelines.xhtml

143 Different independent variables were considered in this study to determine factors associated modern contraceptive utilization. Community level variables, residences, region, and religion were directly accessed from DHS data sets. However, community level poverty, community level education, and community-level media exposure were constructed by aggregating individual-level characteristics at the cluster level. [30] They were categorized as high or low based on the distribution of the proportion values generated for each community after checking the distribution by using the histogram. The aggregate variable was not normally distributed and the median value was used as a cut-off point for the categorization. [30,31] (Table 2)

151 Table 2. List of variables for the assessment of modern contraceptive utilization

Variables	Description
Age of respondents	15-24, 25-34, 35-49
Educational status of respondents	No formal education, Primary education, Secondary and above
Husband education	No formal education, Primary education, Secondary and above
Occupation of respondents	Working, Not working
Husband occupation	Working, Not working
Wealth index	Poor, Middle, Rich
Number of living children	None, 1-4, 5-8, ≥ 9
History of abortion	No, Yes
Births in the last 3 years	No birth, One birth, Two and more birth
Visit of health facility in the last 12 months	Yes, No
Husband's desire for children	Both want the same, Husband wants more, Husband wants fewer, Don't know
Media exposure	No, Yes
Residence	Urban, Rural
Community level poverty	High, Low
Community level media exposure	Low, High
Community level education	Low, High
Region	Somali, Afar, Oromia
Religion	Orthodox Christian, Muslim, Protestant, Others

152

153 Data analysis

For peer review only - http://bmjopen.bmj.com/site/about/guidelines.xhtml

154 For data analysis Stata version 16 software was used. To ensure the representativeness of the EDHS sample and obtain reliable estimations and standard errors, data were weighted (v005/1000000) throughout analysis.

157 Four models fitted: the null model with no explanatory variables, model I with individual factors, model II with community factors, and model III with both individual and community factors. To compare and assess the fitness of nested models, we used the intra class correlation coefficient (ICC), the median odds ratio (MOR), and deviation (-2LLR). Model III was the best-fitting model due to its low deviance. In multivariable analysis, variables with a p-value less than 0.2 in bivariable analysis were used. Finally, in the multivariable analysis, adjusted odds ratios with 95% confidence intervals and p-values less than 0.05 were used to identify factors of modern contraceptive utilization.

165 **Patient and public involvement statement**

166 Reproductive age women were included in this study by providing valuable information. Nevertheless, they have never been involved in the study design, protocol, data collection tools, and reporting disseminating the finding.

For peer review only

For peer review only - http://bmjopen.bmj.com/site/about/guidelines.xhtml

Results

Background characteristics of the study participants

A total weighted sample of 3,822 individual women participated in this study

Out of the total respondents, 2,489 (65.12 %) women were not attended formal education, 2,648 (69.28%) of respondents had no occupation, and 2,761 (72.24%) of the respondents had no media exposure about family planning. Among the participants, 2,071 (54.17%) had 1-4 number of alive children. With regard to their economic status, 1,626 (42.55%) women were from the poor wealth quintiles (Table 3).

Table 3. Individual characteristics of respondents in high fertility regions of Ethiopia (n = 3822)

Variables	Categories	Frequency	Percentage (%)
Age of respondents	15-24	891	23.30
	25-34	1,689	44.19
	35-49	1,242	32.50
Educational status of respondents	No formal education	2,489	65.12
	Primary education	1,204	31.49
	Secondary and higher	130	3.39
Husband education	No formal education	1,795	46.97
	Primary education	1,503	39.33
	Secondary and higher	524	13.70
Occupation of respondents	Not working	2,648	69.28
	Working	1,174	30.72
Husband occupation	Not working	569	14.89
	Working	3,253	85.11
Wealth index	Poor	1,626	42.55
	Middle	749	19.59
	Rich	1,447	37.86
Media exposure	No	2,761	72.24
	Yes	1,061	27.76
Number of living children	None	251	6.56
	1-4	2,071	54.17
	5-8	1,339	35.02

For peer review only - http://bmjopen.bmj.com/site/about/guidelines.xhtml

For peer review only

	≥ 9	162	4.24
History of abortion	No	216	55.18
	Yes	175	44.82
Births in the last 3 years	No birth	1,502	39.28
	One birth	1,885	49.31
	Two and more births	436	11.41
Visit of health facility in the last 12 months	No	2,187	57.22
	Yes	1,635	42.78
Husband's desire for children	Both want the same	1,298	34.03
	Husband wants more	1,162	30.45
	Husband wants fewer	239	6.27
	Don't know	1,116	29.26

Community level factors

Of the respondents 3,390 (88.69%) were rural dwellers. Nearly 60% respondents were Muslims. Among the respondents 3,485 (91.18%) were from Oromia region. About 3,072 (80.38 %) of the respondents were from communities with low proportion of poverty level. Almost two-third (63.24%) of women had media exposure (Table 4).

Table 4. Community level characteristics of respondents in high fertility regions of Ethiopia (n=3822)

Variables	Categories	Frequency	Percentage (%)
Residence	Rural	3,390	88.69
	Urban	432	11.31
Community level poverty	High	750	19.62
	Low	3,072	80.38
Community media exposure	Low	1,405	36.76
	High	2,417	63.24
Community level education	Low	1,214	31.77
	High	2,608	68.23
Region	Afar	82	2.15
	Oromo	3,485	91.18
	Somali	255	6.67
Religion	Orthodox Christian	756	19.77
	Protestant	693	18.13

For peer review only - http://bmjopen.bmj.com/site/about/guidelines.xhtml

	Muslim	2,240	58.61
	Others	133	3.48

Modern contraceptive utilization

Overall, the magnitude of modern contraceptive utilization in high fertility regions of Ethiopia was 29.75 % (95% CI: 28.2, 31.2), with Oromia recording the highest magnitude of 32.18% (Fig. 2).

Multi-level analysis of factors

In terms of the individual level factors, the study showed that reproductive age women aged 25 to 34 and 35 to 49 were more likely to use modern contraceptive (AOR = 1.3; 95% CI: 1.01, 1.66), (AOR = 1.71; 95% CI: 1.37, 2.70) than compared to those aged 15 to 24 women respectively. Women who had a husband with occupation during the time of survey had higher odds (AOR = 1.49; 95% CI 1.03, 1.99) to use modern contraceptive compared to who did not work. Women who had 1-4 number of living children were 2.2 times modern contraceptive use (AOR = 2.20; 95% CI: 1.47, 3.30) than who had no children and reproductive age women who had 5-8 living children were more likely to use modern contraceptive (AOR = 1.74; 95% CI:1.09, 2.77) compared to women who had no living children. Women who had a Partner whose desire for children to have more were 23% less likely to use modern contraceptive (AOR = 0.77; 95% CI: 0.61, 0 .96) compared to both want the same to have children and also women who had a Partner whose desire for children unknown were 29 % less likely to use modern contraceptive (AOR = 0.71; 95% CI: 0.56, 0.89) than both want the same to have children.

Regarding community level factors, the odds of modern contraceptive utilization was high among reproductive age women who had community media exposure (AOR = 1.77; 95% CI:

For peer review only - http://bmjopen.bmj.com/site/about/guidelines.xhtml

1.01, 3.08) compared with their counterparts. Women who were residing in urban area were 2.37 times contraceptive use than living in rural area (AOR = 2.37; 95% CI: 1.20, 4.67). In addition, women in Somali region were 87% less likely (AOR =0.13; 95% CI: 0.03, 0.52) to use modern contraceptive compared to women in Oromia region. Moreover, women who follow Muslim in religion were 51% less likely to use modern contraceptive (AOR =0.49; 95% CI: 0.36, 0.66) than Orthodox Christian followers (Table 5).

Measures of variation

According to the result in the null model, the ICC was 41.1% of the variations of modern contraceptive utilization among study subjects were attributed by difference at the cluster level, but the rest 58.9% were attributed to individual women factors. The final model indicates that the proportional change in variance (PCV) value of 0.606 indicates that both individual and community level factors were responsible for about 60.6% of the variation in modern contraceptive utilization between study subjects. When comparing models/fitness, deviance was used, this model was the best-fitting model, having the lowest deviance (3,551) (Table 5).

Table 5. Multivariable analyses for factors affecting modern contraceptive utilization **(**n=3822).

**Variables**	Model 0	Model 1 AOR (95% CI)	Model 2 AOR (95%CI)	Model 3 AOR (95%CI)
**Individual level Characteristics**				
**Age of respondents**				
15-24		1		1
25-34		1.39 (1.08, 1.77)		**1.29 (1.01, 1.66)***
35-49		0.58 (0.43, 0 .79)		**1.71 (1.37, 2.70)***
**Educational status of the respondents**				
No formal education		1		1

For peer review only - http://bmjopen.bmj.com/site/about/guidelines.xhtml

Primary education	1.15 (0.93, 1.44)		1.06 (0.85, 1.33)
Secondary and higher	1.33(0.78, 2.30)		1.02 (.59, 1.79)
**Husband education**			
No formal education	1		1
Primary education	1.14 (0.93,1.5)		1.05 (0.85, 1.29)
Secondary and higher	1.07 (0.76, 1.47)		0.96 (0.68, 1.34)
**Husband Occupation**			
Not working	1		1
Working	1.55 (1.16, 2.06)		**1.49 (1.03, 1.99)***
**Wealth index**			
Poor	1		1
Middle	1.24 (0.96, 1.59)		1.02 (0.79, 1.31)
Rich	1.53 (1.19, 1.98)		1.19 (0.92, 1.54)
**Media exposure**			
No	1		1
Yes	1.24 (0.99, 1.54)		1.15 (0.92, 1.43)
**Number of living children**			
None	1		1
1-4	2.18 (1.46, 3.25)		**2.20 (1.47, 3.30)***
5-8	1.70 (1.08, 2.69)		**1.74 (1.09, 2.77)***
≥ 9	1.10 (0.51, 2.39)		1.21 (0.55, 2.65)
**Visit of health facility in the last 12 months**			
No	1		1
Yes	1.32 (1.10, 1.58)		1.29 (0.74, 1.54)
**Partner's desire for children**			
Both want the same	1		
Husband wants more	0.75 (0.61, 0.94)		**0.77 (0.61, 0 .96)***
Husband wants fewer	0.84 (0.58, 1.22)		0.82 (0.56, 1.18)
Don't know	0.72 (0.57, 0 .90)		0.71 (0.56, 1.89)
**Community level variables**			
**Residency**			
Rural		1	1
Urban		2.86 (1.48, 5.55)	**2.37 (1.20, 4.67)***
**Community level media exposure**			
Low		1	1
High		1.92 (1.09, 3.39)	**1.77 (1.01, 3.08)***
**Community level poverty**			
High		0.32 (0.15, 0.69	0.33 (0.15, 1.70)
Low		1	**1**
**Community level education**			

For peer review only - http://bmjopen.bmj.com/site/about/guidelines.xhtml

For peer review only

Low			1	1
High			0.81(0.45, 1.43)	0.75 (0.43, 1.31)
**Region**				
Oromo			1	1
Afar			2.25 (0.87, 5.82)	2.19 (0.84, 5.7**)**
Somali			0.11 (0.03, 0.44)	**0.13 (.033, 0.52)***
**Religion**				
Orthodox Christian			1	1
Protestant			1.11 (0.83, 1.50)	1.08 (0.79, 1.47)
Muslim			0.52 (0.38, 0 .71)	**0.49 (0.36, 0.66)***
Others			0.61(0.33, 1.15)	0.61 (0.32, 1.16)
**Random effect results**				
Variance (%)	43.7	32.9	17.8	17.2
ICC (%)	41.1	33.3	20.6	19.5
MOR	17.1	14.8	10.9	10.7
PCV	Ref	24.7	59.3	60.6
**Model comparison**				
Deviance(-2LLR)	3,893	3,747	3,735	3,551

* = P-value < 0.05, ** = P value < 0.01, *** = P value < 0.001 ICC = Intra class correlation coefficient, MOR =Median odds ratio, PCV = proportional change in variance. AOR=adjusted odds ratio; CI= confidence interval.

For peer review only

For peer review only - http://bmjopen.bmj.com/site/about/guidelines.xhtml

Discussion

The aim of this study was to investigate the magnitude and associated factors of modern contraceptive utilization among reproductive age women in high fertility regions of Ethiopia. Slightly above one forth 29.75 % (95% CI: 28.2, 31.2) of participants were currently using modern contraceptive. This study is in line with study conducted in Dembia District.[32] The finding was lower than that reported from 2016 EDHS 35%[33], study done in Adama Town 47% and Ethiopia 51.6%.[34,35] However, this finding is higher than the previous studies conducted in Bale[13] ,Gumuz[36], Rural women in Ethiopia.[37] The difference might be due to the different design and sample size.

In this study, the usage of contraceptives was strongly related with women of reproductive age, specifically those age 25-34 and >35 were more likely to use contraceptives than those age 15-24. The findings were similar to those obtained by Nepal.[18] This may be because younger women (15-24 years old) perceive pregnancy risks are low.

The use of contraception by a wife is determined by her husband, and the wife is more likely to use contraception if her husband approves.[38] In this study, women have a higher likelihood of utilizing contraception if their husband holds a professional, technical, or managerial position. This husband's occupation may reflect husband's educational status and contributed for contraceptive use by women as indicated.[39] There is a higher possibility that these women can reside in urban regions.

The use of contraceptive methods was independently associated with the number of children. In comparison to a woman who had no children, women who had 1-4 children and women who had 5-8 children were nearly 2.2 and nearly 2 times more likely, respectively, to have used a modern form of contraception. Additionally, prior studies have shown that using modern contraceptives

For peer review only

For peer review only - http://bmjopen.bmj.com/site/about/guidelines.xhtml

is more likely when there are a lot of living children.[25,40-44] This might be because women without children might need to have children in order to have the ideal number of children.[45]

The current study also showed that the husband's desire to have more children was inversely related to modern contraceptive use. Women with husbands who want many children were 23% less likely to use modern contraceptives than women who want the same number of children with their husbands. This is congruent with the results of secondary data analyses of the Demographic and Health Survey of Bangladesh, Burkina Faso, and Mali which showed that a husband's desire to have children influenced a woman's use of modern contraception when she was of reproductive age.[46,47]

In regard to community level factors, according to this study, residence was independently associated with modern contraceptive use. Urban women of reproductive age are about three times more likely than rural women to use contemporary contraceptives. This finding is in line with secondary data analysis of Indian, Afghan, Nigerian, and Bangladeshi Demographic and Health Surveys, which showed that urban resident women were more likely than rural resident women to use contemporary contraceptives.[37,48-50] This could be caused by a variety of factors. Urban women tend to be more educated, earn more money, have better access to health facilities, and have better media access than rural women, all of which lead to higher modern contraceptive utilization rates. Women in rural areas also require more children to help them with field work, which negatively impacts their use of modern contraceptives.[29,51-55]

Women who were exposed to the community media had a higher likelihood of using modern methods of contraception than women who weren't. The finding is in line with the results .[25,56] [37,41,43] This is due to the possibility that exposure to mass media could play a significant role in raising awareness and inspiring women to take contemporary contraceptives.

For peer review only - http://bmjopen.bmj.com/site/about/guidelines.xhtml

In addition woman living in Somali region of Ethiopia was 87% less likely to use modern contraceptive compared to women in Oromia region. The reason could be different access to health information and different availability of maternal health services like family planning.[57] Moreover, women who were Muslim religious followers were less likely to use modern contraceptive than Orthodox Christian religious followers. It is possible that the low rate of family planning methods, such as the usage of contemporary contraceptives, among Muslims and Protestants in Ethiopia is the cause of this.[58] In addition, there is evidence that Muslims are less likely to approve of the use of contraceptives and have a negative attitude about family planning.[59]

Strengths and limitations

This study used most recent nationally representative data, which were collected with validated and standardized data collection tools. This also employed multilevel analysis (advanced model) that accounts the correlated nature of EDHS data in the determination of the estimate. Despite the above advantages, the cross sectional nature of the study does not show the cause and effect relationship between the outcome and the independent factors. Moreover, due to EDHS were secondary data, essential factors like attitude and knowledge about contraceptives, partner's perspectives on contraceptives, and fear of side effects were not available in the EDHS.

Conclusion

Modern contraceptives utilization in high fertility regions of Ethiopia was low. Women age, husband occupation, number of living children, husband's desired number of children, residency, community media exposure, region, and religion were significantly associated with modern contraceptive utilization. Therefore, to improve the utilization of modern contraceptives, public

review only

For peer review only - http://bmjopen.bmj.com/site/about/guidelines.xhtml

health policymakers should consider both individual and community level factors when designing family planning programs emphasis should be given to high risk women.

List of abbreviations

AOR: Adjusted Odds Ratio; CI: Confidence interval; EDHS: Ethiopian Demographic and Health Survey; ICC: Intra-class Correlation Coefficient; MOR: Median Odds Ratio; PCV: Proportional Change in Variance

Consent for publication

Not applicable

Availability of data and materials

Data for this study were sourced from Ethiopian Demographic and Health surveys (EDHS), which is freely available online at (https://dhsprogram.com).

Competing interests

The authors declare that they have no competing interests.

Funding

Not applicable

Authors' contributions

TBB conceived the idea, extract the data, data analysis, and draft the manuscript. WDN, DAB, and DBA participate in the data analysis, interpretation, and revising of the manuscript. All authors have read and approved the final manuscript.

Acknowledgments

We are grateful to the EDHS programs, for the permission to use all the relevant EDHS data for this study.

Ethical approval and consent to participate

For peer review only

317 Not applicable/No human participants included. Consent to participants is not applicable since
318 the data is secondary and is available in the public domain. All the methods were conducted
319 according to the Helsinki declarations. More details regarding DHS data and ethical standards
320 are available online at (http://www.dhsprogram.com).

For peer review only

For peer review only - http://bmjopen.bmj.com/site/about/guidelines.xhtml

References

1. Cleland J, Bernstein S, Ezeh A, Faundes A, Glasier A, Innis J. Family planning: the unfinished agenda. The lancet. 2006;368(9549):1810-1827.

2. Kesetebirhan A. National guideline for family planning services in Ethiopia. Federal Democratic Republic of Ethiopia, Ministry of Health. 2011.

3. Nalwadda G, Namutebi M, Volgsten H. Health care providers' perceptions of family planning and contraception education for adolescents in Kampala, Uganda–A qualitative study. Sexual & Reproductive Healthcare. 2019;21:15-20.

4. World Health Organization. Family planning: A global handbook for providers. 2018 Edition. World Health Organization. 2018.

5. Ajong AB, Njotang PN, Kenfack B, et al. Contraceptive method mix and preference: A focus on long acting reversible contraception in urban Cameroon. PloS one. 2018;13(8):e0202967.

6. Creanga AA, Gillespie D, Karklins S, Tsui AO. Low use of contraception among poor women in Africa: an equity issue. Bulletin of the world Health Organization. 2011;89:258-266.

7. Miller K, Miller RA, Askew I, Horn MC, Ndhlovu L. Clinic-based family planning and reproductive health services in Africa: findings from situation analysis studies. 1998.

8. Alenoghena I, Yerumoh S, Momoh A. Knowledge, attitude and uptake of family planning services among women of reproductive age group attending outpatient clinic at a tertiary health institution in Edo State, Nigeria. Journal of Public Health and Epidemiology. 2019;11(3):63-70.

For peer review only - http://bmjopen.bmj.com/site/about/guidelines.xhtml

9. Sharma SK, Pratap N, Ghimire DR. Ethnic differentials of the impact of Family Planning Program on contraceptive use in Nepal. Demographic Research. 2011;25:837-868.

10. Kibria G, Hossen S, Barsha RAA, Sharmeen A, Paul SK, Uddin SI. Factors affecting contraceptive use among married women of reproductive age in Bangladesh. Journal of Molecular Studies and Medicine Research. 2016;2(01):70-79.

11. Peer N, London L, Morojele N. Factors associated with contraceptive use in a rural area in Western Cape Province. South African Medical Journal. 2013;103(6):406-412.

12. Kisia Malalu P, Koskei A, Too R, Chirchir A. Determinants of use of modern family planning methods: A case of Baringo North District, Kenya. 2014.

13. Belda SS, Haile MT, Melku AT, Tololu AK. Modern contraceptive utilization and associated factors among married pastoralist women in Bale eco-region, Bale Zone, South East Ethiopia. BMC health services research. 2017;17(1):1-12.

14. Mekonnen W, Worku A. Determinants of low family planning use and high unmet need in Butajira District, South Central Ethiopia. Reproductive health. 2011;8(1):1-8.

15. Lakew Y, Reda AA, Tamene H, Benedict S, Deribe K. Geographical variation and factors influencing modern contraceptive use among married women in Ethiopia: evidence from a national population based survey. Reproductive health. 2013;10(1):1-10.

16. Tilahun T, Coene G, Luchters S, et al. Family planning knowledge, attitude and practice among married couples in Jimma Zone, Ethiopia. PloS one. 2013;8(4):e61335.

17. Adebowale SA, Palamuleni ME. Determinants of unmet need for modern contraception and reasons for non-use among married women in rural areas of Burkina Faso. African Population Studies. 2014:499-514.

For peer review only

For peer review only - http://bmjopen.bmj.com/site/about/guidelines.xhtml

365 18. Fotso JC, Izugbara C, Saliku T, Ochako R. Unintended pregnancy and subsequent use of modern contraceptive among slum and non-slum women in Nairobi, Kenya. BMC pregnancy and childbirth. 2014;14(1):1-10.

368 19. Apanga PA, Adam MA. Factors influencing the uptake of family planning services in the Talensi District, Ghana. Pan African Medical Journal. 2015;20(1).

370 20. Medhanyie AA, Desta A, Alemayehu M, et al. Factors associated with contraceptive use in Tigray, North Ethiopia. Reproductive health. 2017;14(1):1-11.

372 21. Mohammed A, Woldeyohannes D, Feleke A, Megabiaw B. Determinants of modern contraceptive utilization among married women of reproductive age group in North Shoa Zone, Amhara Region, Ethiopia. Reproductive health. 2014;11(1):1-7.

375 22. Kahsay ZH, Hiluf MK, Shamie R, Tadesse Y, Bazzano AN. Pregnant Women's intentions to deliver at a health Facility in the Pastoralist Communities of Afar, Ethiopia: an application of the health belief model. International Journal of Environmental Research and Public Health. 2019;16(5):888.

379 23. Sedlander E, Bingenheimer JB, Edberg MC, Rimal RN, Shaikh H, Munar W. Understanding modern contraception uptake in one Ethiopian community: a case study. Reproductive health. 2018;15(1):1-8.

382 24. Fantay Gebru K, Mekonnen Haileselassie W, Haftom Temesgen A, Oumer Seid A, Afework Mulugeta B. Determinants of stunting among under-five children in Ethiopia: a multilevel mixed-effects analysis of 2016 Ethiopian demographic and health survey data. BMC pediatrics. 2019;19(1):1-13.

For peer review only - http://bmjopen.bmj.com/site/about/guidelines.xhtml

25. Abate MG, Tareke AA. Individual and community level associates of contraceptive use in Ethiopia: a multilevel mixed effects analysis. Archives of Public Health. 2019;77(1):1-12.

26. African countries with the highest fertility rate | Statista https://worldpopulationreview.com/countries/total-fertility-rate. cited on December 8, 2021.

27. Central statistical agency (CSA) [Ethiopia] and ICF. Ethiopia demographic and health survey, Addis Ababa, Ethiopia and Calverton, Maryland, USA. 2016.

28. Corsi DJ, Neuman M, Finlay JE, Subramanian S. Demographic and health surveys: a profile. International journal of epidemiology. 2012;41(6):1602-1613.

29. Gebre MN, Edossa ZK. Modern contraceptive utilization and associated factors among reproductive-age women in Ethiopia: evidence from 2016 Ethiopia demographic and health survey. BMC Women's Health. 2020;20(1):1-14.

30. Liyew AM, Teshale AB. Individual and community level factors associated with anemia among lactating mothers in Ethiopia using data from Ethiopian demographic and health survey, 2016; a multilevel analysis. BMC Public Health. 2020;20(1):1-11.

31. Getaneh T, Negesse A, Dessie G, Desta M, Moltot T. Predictors of unmet need for family planning in Ethiopia 2019: a systematic review and meta analysis. Archives of Public Health. 2020;78(1):1-11.

32. Debebe S, Limenih MA, Biadgo B. Modern contraceptive methods utilization and associated factors among reproductive aged women in rural Dembia District, northwest Ethiopia: Community based cross-sectional study. International Journal of Reproductive BioMedicine. 2017;15(6):367.

For peer review only

For peer review only - http://bmjopen.bmj.com/site/about/guidelines.xhtml

33. Ethiopia Demographic Health Survey-2011. Central Statistical Agency Addis Ababa. Ethiopia ICF International Calverton, Maryland, USA. 20122016.

34. Biruk T, Assefa H, Georges R. The prevalence of Covert use of contraceptives in Nazareth/Adama town. Eur J Contracept Reprod Health Care. 2008;13(2):63.

35. Abraha MW, Nigatu TH. Modeling trends of health and health related indicators in Ethiopia (1995-2008): a time-series study. Health Research Policy and Systems. 2009;7(1):1-17.

36. Adane AA, Bekele YA, Melese E, Worku GT, Netsere HB. Modern contraceptive utilization and associated factors among married gumuz women in Metekel Zone North West Ethiopia. BioMed Research International. 2020;2020.

37. Fenta SM, Gebremichael SG. Predictors of modern contraceptive usage among sexually active rural women in Ethiopia: A multi-level analysis. Archives of Public Health. 2021;79(1):1-10.

38. Kamal N. The influence of husbands on contraceptive use by Bangladeshi women. Health policy and planning. 2000;15(1):43-51.

39. Gubhaju B. The influence of wives' and husbands' education levels on contraceptive method choice in Nepal, 1996-2006. International Perspectives on Sexual and Reproductive Health. 2009:176-185.

40. Idris H. Factors Affecting the Use of Contraceptive in Indonesia: Analysis from the National Socioeconomic Survey (Susenas). KEMAS: Jurnal Kesehatan Masyarakat. 2019;15(1):117-123.

For peer review only - http://bmjopen.bmj.com/site/about/guidelines.xhtml

41. Rutaremwa G, Kabagenyi A, Wandera SO, Jhamba T, Akiror E, Nviiri HL. Predictors of modern contraceptive use during the postpartum period among women in Uganda: a population-based cross sectional study. BMC public health. 2015;15(1):1-9.

42. Musa A, Assefa N, Weldegebreal F, Mitiku H, Teklemariam Z. Factor associated with experience of modern contraceptive use before pregnancy among women who gave birth in Kersa HDSS, Ethiopia. BMC public health. 2016;16(1):1-6.

43. Nsanya MK, Atchison CJ, Bottomley C, Doyle AM, Kapiga SH. Modern contraceptive use among sexually active women aged 15–19 years in North-Western Tanzania: results from the Adolescent 360 (A360) baseline survey. BMJ open. 2019;9(8):e030485.

44. Devita VD, Rosliza A, Suriani I. Prevalence of modern contraceptive use among reproductive women with hypertension and diabetes in a government hospital in BATAM, Indonesia and its socio-demographic determinants. International Journal of Public Health and Clinical Sciences. 2018;5(4):297-278.

45. Matovu JK, Makumbi F, Wanyenze RK, Serwadda D. Determinants of fertility desire among married or cohabiting individuals in Rakai, Uganda: a cross-sectional study. Reproductive health. 2017;14(1):1-11.

46. O'Regan A, Thompson G. Indicators of young women's modern contraceptive use in Burkina Faso and Mali from Demographic and Health Survey data. Contraception and reproductive medicine. 2017;2(1):1-8.

47. Hossain M, Khan M, Ababneh F, Shaw J. Identifying factors influencing contraceptive use in Bangladesh: evidence from BDHS 2014 data. BMC public health. 2018;18(1):1-14.

For peer review only

For peer review only - http://bmjopen.bmj.com/site/about/guidelines.xhtml

48. Ezeh OK, Agho KE, Dibley MJ, Hall J, Page AN. The impact of water and sanitation on childhood mortality in Nigeria: evidence from demographic and health surveys, 2003–2013. International journal of environmental research and public health. 2014;11(9):9256-9272.

49. Haq I, Sakib S, Talukder A. Sociodemographic factors on contraceptive use among ever-married women of reproductive age: evidence from three demographic and health surveys in Bangladesh. Medical Sciences. 2017;5(4):31.

50. Dey AK. Socio-demographic determinants and modern family planning usage pattern-an analysis of National Family Health Survey-IV data. Int J Community Med Public Heal. 2019;6(2):738.

51. Tekelab T, Melka AS, Wirtu D. Predictors of modern contraceptive methods use among married women of reproductive age groups in Western Ethiopia: a community based cross-sectional study. BMC women's health. 2015;15(1):1-8.

52. Johnson OE. Determinants of modern contraceptive uptake among Nigerian women: evidence from the national demographic and health survey. African journal of reproductive health. 2017;21(3):89-95.

53. Osmani AK, Reyer JA, Osmani AR, Hamajima N. Factors influencing contraceptive use among women in Afghanistan: secondary analysis of Afghanistan Health Survey 2012. Nagoya journal of medical science. 2015;77(4):551.

54. Adebowale AS, Gbadebo B, Afolabi FR. Wealth index, empowerment and modern contraceptive use among married women in Nigeria: are they interrelated? Journal of Public Health. 2016;24(5):415-426.

For peer review only - http://bmjopen.bmj.com/site/about/guidelines.xhtml

55. Adebowale SA, Adedini SA, Ibisomi LD, Palamuleni ME. Differential effect of wealth quintile on modern contraceptive use and fertility: evidence from Malawian women. BMC women's health. 2014;14(1):1-13.

56. Agbadi P, Tagoe E, Akosua AF, Owusu S. A multilevel analysis of predictors of modern contraceptive use among reproductive age women in Sierra Leone: insight from demographic and Health surveys: Center for Open Science;2019.

57. Ababa A. Ethiopia. Abstract available from: https://wfpha. confex. com/wfpha/2012/webprogram. Paper10587. html [Google Scholar]. 2013.

58. Roudi-Fahimi F. Islam and family planning: Population Reference Bureau Washington, DC; 2004.

59. Duze MC, Mohammed IZ. Male knowledge, attitude, and family planning practices in Northern Nigeria. African Journal of Reproductive Health. 2006;10(3):53-65.

For peer review only

For peer review only - http://bmjopen.bmj.com/site/about/guidelines.xhtml

487 **List of Legends**

488 Figure 1. Schematic illustration of women included in high fertility regions of Ethiopia

489 Figure 2. Magnitude of modern contraceptive utilization in high fertility regions of Ethiopia

For peer review only

For peer review only - http://bmjopen.bmj.com/site/about/guidelines.xhtml

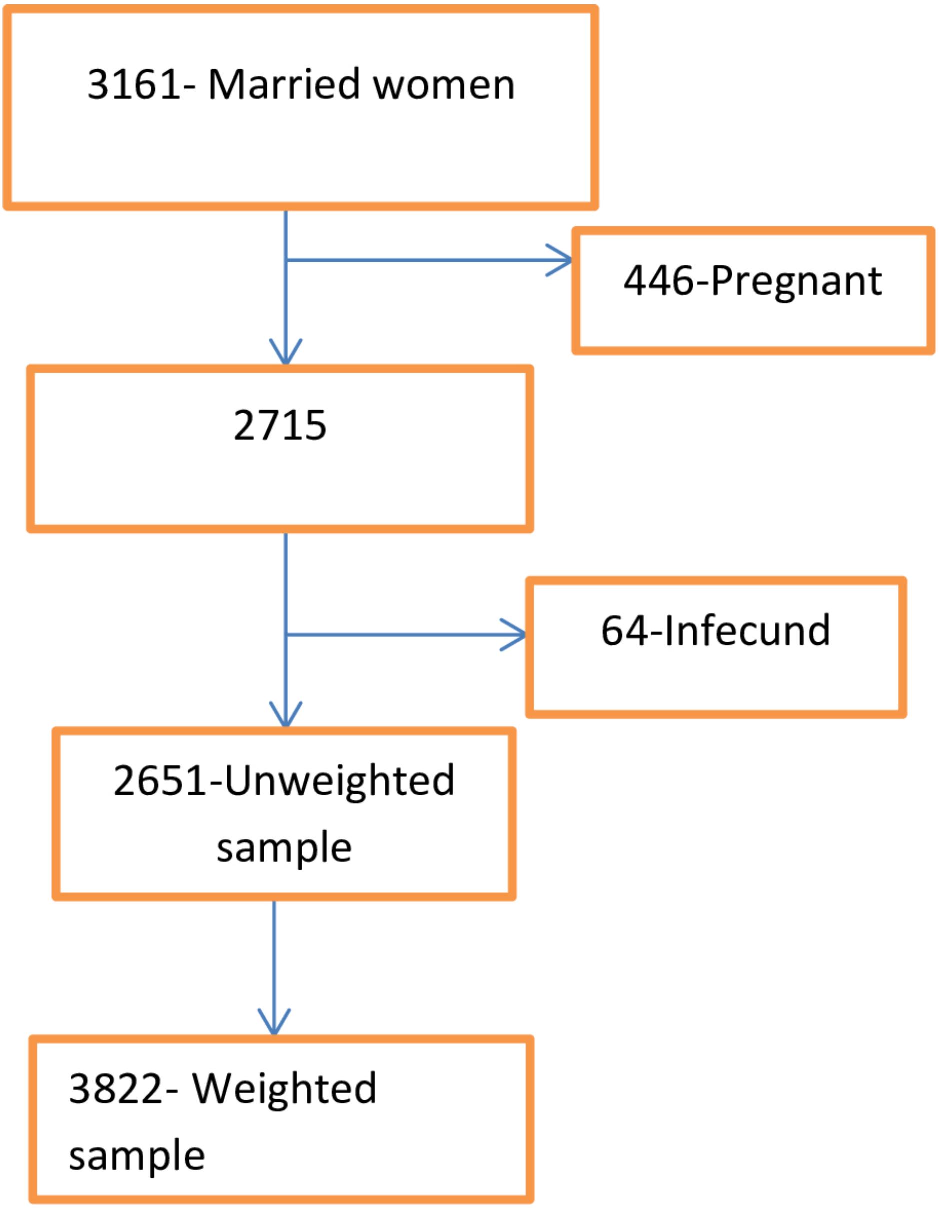

Figure 1. Schematic illustration of women included in high fertility regions of Ethiopia

77x98mm (300 x 300 DPI)

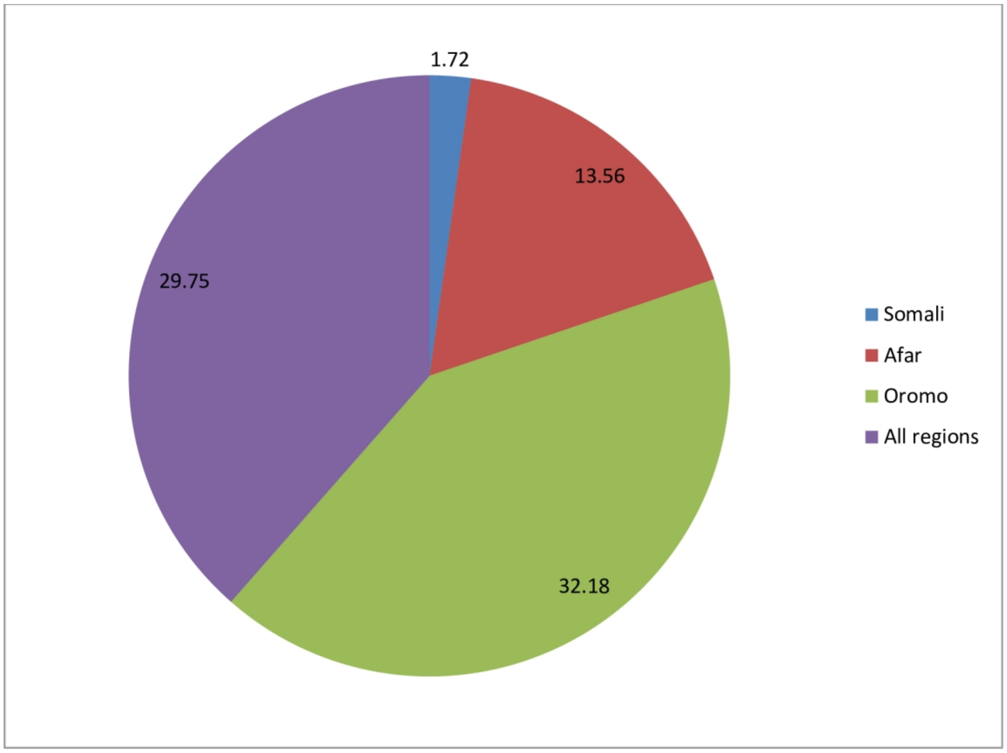

Figure 2. Magnitude of modern contraceptive utilization in high fertility regions of Ethiopia

156x116mm (300 x 300 DPI)

STROBE Statement—Checklist of items that should be included in reports of ***cross-sectional studies***

**Items**	**Number**	**Recommendations**
**Title and abstract**	1	(*a*) Indicate the study's design with a commonly used term in the title or the abstract **page 2, line number 24**
		(b) provide in the abstract an informative and balanced summary of what was done and what was found **Page 2, line number 27-39**
**Introduction**		
Background/rational	2	Explain the scientific background and rationale for the investigation being reported **Page 4-5, line number 59-100**
Objective	3	State specific objectives, including any prespecified hypotheses **Page 2, line number 22-23**
**Methods**		
Study design	4	Present key elements of study design early in the paper **page 6, line number 103-104**
Setting	5	Describer the setting, location, and relevant dates including period of recruitment and data collection **Page number 6, line number 108-111**

For peer review only

Participants	6	Give the eligibility criteria, and the sources and methods of selection of participants<br>**Page 2, line number 26**
		Clearly define all outcomes, exposures, predictors, potential confounders, and effect modifier, Give diagnostic, if applicable<br>**Page 7-8 line number 131-147**
Data source and measurement	7	**Page 6-7 line number 116-128**
Bias	8	Describe any efforts to address potential sources of bias<br>**N/A**
Study size	9	Explain how the study design was arrived<br>**Page 6 , line number 105-108**
Quantitative variables	10	Explain how quantitative variables were handled in the analysis , if applicable describe which groupings chose and why<br>**Page7 -8, line number 131-147**
Statistical methods	11	(a)Describe all statistical methods, including those used to control for confounding<br>**Page 8, line number 148-159**

For peer review only

		(*b*) Describe any methods used to examine subgroups and interactions<br>**NA**
		(*c*) Explain how missing data were addressed<br>**NA**
		(*d*) If applicable, describe analytical methods taking account of sampling strategy<br>**NA**
		(*e*) Describe any sensitivity analyses<br>**NA**
**Results**		
Descriptive data	12	Give characteristics of study participants (eg, demographic, clinical, social) and information on exposure and potential confounders<br>**Page 9, line number 171-181**
Outcome data	13	Report numbers of outcome events or summary measures<br>**Page 10, line number 183-184**
Main results	14	(a) Give unadjusted estimates and, if applicable, confounder adjusted estimates and their precision (e.g., 95% confidence interval).Make clear which confounders were adjusted for and why they were included<br>**Page 9-10, line number 187-207**

For peer review only

		(b) If relevant, consider translating estimates of relative risk into absolute risk for a meaningful time period<br>**NA**
Other analysis	15	Report other analyses doing analyses of subgroups and interactions, and sensitive analysis<br>**NA**
**Discussion**		
Key result	16	Summaries key results with reference to study objectives<br>**Page 12, line number 119-224**
Limitations	17	Discuss limitations of the study, taking into account sources of potential bias or imprecision. discuss both direction and magnitude of any bias<br>**Page 14 , page 274-278**
Interpretation	18	Give a cautious overall interpretation of results considering objectives, limitations, multiplicity of analysis, result from similar studies, and other relevant evidence<br>**Page 12-14, line number 217-278**
Generalizability	19	Discuss the generalizability (external validity) of the study results<br>**Page14-15, line number 280-285**

For peer review only

Funding	20	Give the source of funding and the role of the funders for the present study and if applicable for the original study on which the present article is based<br>**Page 15, line number 298**

Note: An explanation and elaboration article discusses each checklist item and gives methodological background published examples of transport reporting. The STROBE checklist is best used in conjunction with this articles (freely available on the web sites of PloS Medicine at http://www.plosmedicene.org/.Annals of internal medicine http://www.annals.org/, and Epidemiology at http://www.epidem.com/ information on the STROBE initiative is available at www.strobe-statement.org.

For peer review only

BMJ Open

Modern contraceptive utilization and its associated factors among reproductive age women in high fertility regions of Ethiopia: A multilevel analysis of Ethiopia Demographic and Health Survey

Journal:	*BMJ Open*
Manuscript ID	bmjopen-2022-066432.R1
Article Type:	Original research
Date Submitted by the Author:	18-Jan-2023
Complete List of Authors:	Belachew, Tadele; University of Gondar, Department of Health Systems and Policy<br>Negash, Wubshet Debebe; University of Gondar, Department of Health Systems and Policy<br>Bitew, Desalegn Anmut; University of Gondar, Department of Reproductive Health, Institute of Public Health<br>Asmamaw, Desale; University of Gondar, Department of Reproductive Health, Institute of Public Health
**Primary Subject Heading**:	Health policy
Secondary Subject Heading:	Health services research
Keywords:	Nutrition < TROPICAL MEDICINE, Epidemiology < TROPICAL MEDICINE, Economics < TROPICAL MEDICINE, Health policy < HEALTH SERVICES ADMINISTRATION & MANAGEMENT, EPIDEMIOLOGY

SCHOLARONE™
Manuscripts

I, the Submitting Author has the right to grant and does grant on behalf of all authors of the Work (as defined in the below author licence), an exclusive licence and/or a non-exclusive licence for contributions from authors who are: i) UK Crown employees; ii) where BMJ has agreed a CC-BY licence shall apply, and/or iii) in accordance with the terms applicable for US Federal Government officers or employees acting as part of their official duties; on a worldwide, perpetual, irrevocable, royalty-free basis to BMJ Publishing Group Ltd ("BMJ") its licensees and where the relevant Journal is co-owned by BMJ to the co-owners of the Journal, to publish the Work in this journal and any other BMJ products and to exploit all rights, as set out in our licence.

The Submitting Author accepts and understands that any supply made under these terms is made by BMJ to the Submitting Author unless you are acting as an employee on behalf of your employer or a postgraduate student of an affiliated institution which is paying any applicable article publishing charge ("APC") for Open Access articles. Where the Submitting Author wishes to make the Work available on an Open Access basis (and intends to pay the relevant APC), the terms of reuse of such Open Access shall be governed by a Creative Commons licence – details of these licences and which Creative Commons licence will apply to this Work are set out in our licence referred to above.

Other than as permitted in any relevant BMJ Author's Self Archiving Policies, I confirm this Work has not been accepted for publication elsewhere, is not being considered for publication elsewhere and does not duplicate material already published. I confirm all authors consent to publication of this Work and authorise the granting of this licence.

For peer review only

Modern contraceptive utilization and its associated factors among reproductive age women in high fertility regions of Ethiopia: A multilevel analysis of Ethiopia Demographic and Health Survey

Tadele Biresaw Belachew[1*], Wubshet Debebe Negash[1], Desalegn Anmut Bitew[2], Desale Bihonegn Asmamaw[2]

[1]Department of Health Systems and Policy, Institute of Public Health, College of Medicine and Health Sciences, University of Gondar, Gondar, Ethiopia

[2]Department of Reproductive Health, Institute of Public Health, College of Medicine and Health Sciences, University of Gondar, Gondar, Ethiopia

Authors' addresses

TBB: tadelebiresaw01@gmail.com

WDN: wubshetdn@gmail.com

DAB: Desalegn1227@gmail.com

DBA: desalebihonegn1988@gmail.com

Corresponding author:

Tadele Biresaw Belachew (TBB)

Email: tadelebiresaw01@gmail.com

Phone: +251-918747633

P.O.Box: 196; University of Gondar, Gondar, Ethiopia

For peer review only

For peer review only - http://bmjopen.bmj.com/site/about/guidelines.xhtml

Abstract

Objective: This study is aimed to assess the magnitude of modern contraceptives utilization and associated factors among reproductive age women in high fertility regions of Ethiopia

Design: Cross-sectional study

Setting: High fertility regions of Ethiopian

Participants: A total weighted sample of 3,822 married reproductive age women

Methods: In this study, data were obtained from the recent Demographic and Health Surveys (EDHS). A total weighted sample of 3,822 women of reproductive age was included. A multilevel mixed-effect binary logistic regression model was fitted to identify the significant associated factors of modern contraceptive utilization. Statistical significance was determined using Adjusted Odds Ratio (AOR) with 95% confidence interval.

Results: The overall modern contraceptive utilization was 29.75% (95% CI: 28.2, 31.2). Among the factors associated with utilization were women's age 25-34 years (AOR = 1.3; 95% CI: 1.01, 1.66), and ≥35 (AOR = 1.71; 95% CI: 1.37, 2.70), husband's occupation (AOR = 1.49; 95% CI 1.03, 1.99), number of alive children: 1-4 (AOR = 2.20; 95% CI: 1.47, 3.30), 5-8 (AOR = 1.74; 95% CI:1.09, 2.77), husband's desired number of children (AOR = 0.77; 95% CI: 0.61, 0 .96), residency (AOR = 2.37; 95% CI: 1.20, 4.67), community media exposure (AOR = 1.77; 95% CI: 1.01, 3.08), region (AOR =0.13; 95% CI: 0.03, 0.52), and religion (AOR =0.49; 95% CI: 0.36, 0.66) were significantly associated with modern contraceptive utilization.

Conclusion: Modern contraceptives utilization in high fertility regions of Ethiopia was low. Women age, husband occupation, number of living children, husband's desired number of children, residency, community media exposure, region, and religion were significantly

For peer review only

associated with modern contraceptive utilization. Therefore, to improve the utilization of modern contraceptives, public health policymakers should consider creating awareness through mass media, male involvement in family planning, as well as family planning programs, should be encouraged in rural areas.

Keywords: Modern contraceptive, multilevel, factors, Ethiopia

Strengths and limitation of study

- This study used most recent nationally representative data, which were collected validated and standard data collection tools.
- This study employed multilevel analysis (advanced model) that accounts the correlated nature of EDHS data in the determination of the estimate.
- The cross sectional nature of the study does not show the cause and effect relationship between the outcome and the independent factors.
- Moreover, due to EDHS were secondary data, essential factors like attitude and knowledge about contraceptives, partner's perspectives on contraceptives, and fear of side effects were not available in the EDHS

For peer review only

Background

Family Planning services support people in making decisions about whether to have children by teaching, advocating, and offering birth control methods.[1] It also prevents pregnancy-related health risks in women, infant mortality, sexually transmitted infections (STIs), and adolescent pregnancy, while slowing population growth.[2,3] Additionally, it is the best investment in the health care and wellbeing of women, children, and communities.[2] Access to quality, affordable sexual and reproductive health services like contraceptives and information is crucial to ensure the rights and wellbeing of women and girls.[4]

The types of contraceptive methods used for family planning can be divided into modern contraception and traditional methods that limit or delay childbirth.[5] Modern contraceptive methods are more effective than traditional methods that include short-acting (injectable, pills, male condom, female condom, emergency contraceptives) [6-8], long-acting reversible (hormonal implants and intrauterine devices (IUD)) [9-11], and permanent (tubal-ligation (female sterilization) and vasectomy (male sterilization)) [12-14] methods that exclude traditional methods which helps to limit number of children and prevent maternal and child morbidity and mortality.[15]

In spite of contraceptive value in improving maternal and child health, a large proportion of women do not use contraceptives with remarkable variations across geographical areas and within country. Though, in the past twenty years, the utilization of modern contraceptives has increased slightly, from 54.4% in 20th to 57.4% in 21th in the world and 23.6% in 2008 to 28.5% in 2015 in Africa respectively, as reported by the United Nations Population Fund (UNFPA).[16,17]

For peer review only - http://bmjopen.bmj.com/site/about/guidelines.xhtml

According to studies conducted in Asia, modern contraceptive use among married women in Nepal and Bangladesh was 36% and 81.27%, respectively.[18,19] similarly, studies in Africa show the high levels of modern contraception utilization in South Africa, Baringo Kenya, and Nigeria at 39.9%, 32%, 31%, and 92.7%, respectively.[17,20,21] Moreover, different studies conducted in Ethiopia also shows that the magnitude of modern contraceptive utilization ranges from 9.1%-46.9%.[22-25]

Based on studies conducted in developing countries, factors affecting the use of contraceptives included age, education level, parity, religion, knowledge about modern family planning methods and side effects, method approval by partners, and employment status.[26-28] In addition, studies in Ethiopia found that age, residence, education level of the mother, couple discussions, perception of husband approval, discussions with health extension workers, psychological acceptance, desire for more children, monthly income, and number of living children were all affected by modern family planning method use.[22,24,29,30]

In Ethiopia, a variety of strategies have been employed to increase the uptake of contraceptive methods over the last decade. Among the steps taken to increase contraceptive use was the implementation of health extension programs to change attitudes and improve awareness among the community.[31,32] To remove health system barriers to the use of contraceptives, the health system expanded health centers and health posts and upgraded primary health care units.[31] In spite of these efforts made at the national level, the proportion of women who use contraceptive methods remains low.[33]

We therefore aimed to determine what factors are associated with use of modern contraceptives among women of reproductive age groups in high fertility areas of Ethiopia. It is hoped that the

For peer review only

For peer review only - http://bmjopen.bmj.com/site/about/guidelines.xhtml

results of the study will help policy makers to make interventions that will help reduce maternal mortality and morbidity through speeding up the utilization of modern contraceptives.

For peer review only

For peer review only - http://bmjopen.bmj.com/site/about/guidelines.xhtml

Methods

Study design, period, and setting

A community-based cross-sectional survey was conducted using secondary data in the 2016 Ethiopian Demographic and Health Surveys (EDHS)[34] which was conducted by the Central Statistical Agency (CSA) in collaboration with the Federal Ministry of Health (FMoH) and the Ethiopian Public Health Institute (EPHI), which was a national representative sample conducted from January 18 to June 27, 2016. There are nine regional states in Ethiopia (Tigray, Afar, Amhara, Oromia, Benishangul, Gambela, South Nation, Nationalities and Peoples' Region (SNNPR), Harari, and Somali), and two administrative cities (Addis Ababa and Dire-Dawa), 611 Districts, and 15,000 Kebeles. Afar, Somali and Oromia regions were included in this study. These regions were selected because they are the high fertility rate regions in Ethiopia with fertility rates above 5.0, a value that is higher than the rate of 4.6 in Ethiopia and 2.47 worldwide.[35,36]

Data sources

The data for these regions were gained from the official database of the DHS program, www.measuredhs.com after authorization was granted via online request by explaining the purpose of our study. We extracted dependent and independent variables from the woman record (IR file). DHS is a nationally representative household survey conducted by face-to-face interviews on a wide range of populations. Study participants were selected using a two-stage stratified sampling technique. Enumeration Areas (EAs) were randomly selected in the first stage, while households were selected in the second stage .[37] We calculated the individual weight for women (v005) by multiplying the household weight (hv005) by the inverse of the individual response rate. Before analysis, individual sample weights are generated by dividing (v005) by

For peer review only

For peer review only - http://bmjopen.bmj.com/site/about/guidelines.xhtml

127 one million to approximate the number of cases.[10,38,39] After exclusion of pregnant women and
128 infecund during survey, a total weighted sample of 3,822 married reproductive- age women were
129 included from three regions in this study (Fig. 1).

130 **Variables and measurements**

131 **Dependent variable**

132 The outcome variable was modern contraceptive utilization. In the current study a woman was
133 considered as modern contraceptive method utilizer if she had been using at least one of the
134 modern contraceptives (female sterilization, male sterilization, IUCD, injectable, implants, pills,
135 male condom, female condom, and emergency contraception) during EDHS data collection
136 period. Whereas a woman was considered to be non-utilizer of the modern contraceptive method
137 if she had been using traditional methods like rhythm method, lactation amenorrhea method, and
138 withdrawal or if she had not been using any type of contraception during EDHS data collection
139 period.[40]

140 **Independent variables**

141 Different independent variables were considered in this study to determine factors associated
142 modern contraceptive utilization. Community level variables, residences, region, distance to the
143 health facilities, and religion were directly accessed from EDHS data sets. However, community
144 level poverty, community level education, and community-level media exposure were
145 constructed by aggregating individual-level characteristics at the cluster level. [41] They were
146 categorized as high or low based on the distribution of the proportion values generated for each
147 community after checking the distribution by using the histogram. The aggregate variable was
148 not normally distributed and the median value was used as a cut-off point for the categorization.
149 [41,42] (Table 1)

For peer review only - http://bmjopen.bmj.com/site/about/guidelines.xhtml

Table 1. List of variables for the assessment of modern contraceptive utilization

Variables	Description
Age of respondents	15-24, 25-34, 35-49
Educational status of respondents	No formal education, Primary education, Secondary and above
Husband education	No formal education, Primary education, Secondary and above
Occupation of respondents	Working, Not working
Husband occupation	Working, Not working
Wealth index	Poor, Middle, Rich
Number of living children	None, 1-4, 5-8, ≥ 9
History of abortion	No, Yes
Births in the last 3 years	No birth, One birth, Two and more birth
Visit of health facility in the last 12 months	Yes, No
Husband's desire for children	Both want the same, Husband wants more, Husband wants fewer, Don't know
Media exposure	No, Yes
Residence	Urban, Rural
Community level poverty	High, Low
Community level media exposure	Low, High
Community level education	Low, High
Region	Somali, Afar, Oromia
Religion	Orthodox Christian, Muslim, Protestant, Others
Distance to the health facilities	Big problem, Not big problem

Data analysis

For data analysis Stata version 16 software was used. To ensure the representativeness of the EDHS sample and obtain reliable estimations and standard errors, data were weighted (v005/1000000) throughout analysis.

Four models fitted: the null model with no explanatory variables, model I with individual factors, model II with community factors, and model III with both individual and community factors. To compare and assess the fitness of nested models, we used the intra class correlation coefficient (ICC), the median odds ratio (MOR), and deviation (-2LLR). Model III was the best-fitting model due to its low deviance. In multivariable analysis, variables with a p-value less than 0.2 in

For peer review only - http://bmjopen.bmj.com/site/about/guidelines.xhtml

161 bivariable analysis were used. Finally, in the multivariable analysis, adjusted odds ratios with 95% confidence intervals and p-values less than 0.05 were used to identify factors of modern contraceptive utilization.

Ethical approval

Not applicable/No human participants included. Consent to participants is not applicable since the data is secondary and is available in the public domain. All the methods were conducted according to the Helsinki declarations. More details regarding DHS data and ethical standards are available online at (http://www.dhsprogram.com).

Patient and public involvement statement

No patient or the public was directly involved in developing the research questions, the design, protocol, data collection tools, results and dissemination plan of the study.

For peer review only

For peer review only - http://bmjopen.bmj.com/site/about/guidelines.xhtml

Results

Background characteristics of the study participants

A total weighted sample of 3,822 individual women participated in this study.

Out of the total respondents, 2,489 (65.12 %) women were not attended formal education, 2,648 (69.28%) of respondents had no occupation, and 2,761 (72.24%) of the respondents had no media exposure about family planning. Among the participants, 2,071 (54.17%) had 1-4 number of alive children. With regard to their economic status, 1,626 (42.55%) women were from the poor wealth quintiles (Table 2).

For peer review only

Table 2. Individual characteristics of respondents in high fertility regions of Ethiopia (n = 3822)

**Variables**	**Categories**	**Frequency**	**Percentage (%)**
Age of respondents	15-24	891	23.30
	25-34	1,689	44.19
	35-49	1,242	32.50
Educational status of respondents	No formal education	2,489	65.12
	Primary education	1,204	31.49
	Secondary and higher	130	3.39
Husband education	No formal education	1,795	46.97
	Primary education	1,503	39.33
	Secondary and higher	524	13.70
Occupation of respondents	Not working	2,648	69.28
	Working	1,174	30.72
Husband occupation	Not working	569	14.89
	Working	3,253	85.11
Wealth index	Poor	1,626	42.55
	Middle	749	19.59
	Rich	1,447	37.86
Media exposure	No	2,761	72.24
	Yes	1,061	27.76
Number of living children	None	251	6.56
	1-4	2,071	54.17
	5-8	1,339	35.02

For peer review only - http://bmjopen.bmj.com/site/about/guidelines.xhtml

	≥ 9	162	4.24
History of abortion	No	216	55.18
	Yes	175	44.82
Births in the last 3 years	No birth	1,502	39.28
	One birth	1,885	49.31
	Two and more births	436	11.41
Visit of health facility in the last 12 months	No	2,187	57.22
	Yes	1,635	42.78
Husband's desire for children	Both want the same	1,298	34.03
	Husband wants more	1,162	30.45
	Husband wants fewer	239	6.27
	Don't know	1,116	29.26

Community level factors

Of the respondents 3,390 (88.69%) were rural dwellers. Nearly 60% respondents were Muslims. Among the respondents 3,485 (91.18%) were from Oromia region. About 3,072 (80.38 %) of the respondents were from communities with low proportion of poverty level. Almost two-third (63.24%) of women had media exposure and 2,235 (58.48%) of participant's perceived as big problem to access health facilities (Table 3).

Table 3. Community level characteristics of respondents in high fertility regions of Ethiopia (n=3822)

**Variables**	**Categories**	**Frequency**	**Percentage (%)**
Residence	Rural	3,390	88.69
	Urban	432	11.31
Community level poverty	High	750	19.62
	Low	3,072	80.38
Community media exposure	Low	1,405	36.76
	High	2,417	63.24
Community level education	Low	1,214	31.77
	High	2,608	68.23
Region	Afar	82	2.15
	Oromo	3,485	91.18
	Somali	255	6.67
Religion	Orthodox Christian	756	19.77

For peer review only - http://bmjopen.bmj.com/site/about/guidelines.xhtml

	Protestant	693	18.13
	Muslim	2,240	58.61
	Others	133	3.48
Distance to health facilities	Big problem	2,235	58.48
	Not big problem	1,587	41.52

Modern contraceptive utilization

Overall, the magnitude of modern contraceptive utilization in high fertility regions of Ethiopia was 29.75 % (95% CI: 28.2, 31.2), with Oromia recording the highest magnitude of 32.18% (Fig. 2).

Multi-level analysis of factors

In terms of the individual level factors, the study showed that reproductive age women aged 25 to 34 and 35 to 49 were more likely to use modern contraceptive (AOR = 1.3; 95% CI: 1.01, 1.66), (AOR = 1.71; 95% CI: 1.37, 2.70) than compared to those aged 15 to 24 women respectively. Women who had a husband with occupation during the time of survey had higher odds (AOR = 1.49; 95% CI 1.03, 1.99) to use modern contraceptive compared to who did not work. Women who had 1-4 number of living children were 2.2 times modern contraceptive use (AOR = 2.20; 95% CI: 1.47, 3.30) than who had no children and reproductive age women who had 5-8 living children were more likely to use modern contraceptive (AOR = 1.74; 95% CI:1.09, 2.77) compared to women who had no living children. Women who had a Partner whose desire for children to have more were 23% less likely to use modern contraceptive (AOR = 0.77; 95% CI: 0.61, 0 .96) compared to both want the same to have children and also women who had a Partner whose desire for children unknown were 29 % less likely to use modern contraceptive (AOR = 0.71; 95% CI: 0.56, 0.89) than both want the same to have children.

For peer review only - http://bmjopen.bmj.com/site/about/guidelines.xhtml

Regarding community level factors, the odds of modern contraceptive utilization was high among reproductive age women who had community media exposure (AOR = 1.77; 95% CI: 1.01, 3.08) compared with their counterparts. Women who were residing in urban area were 2.37 times contraceptive use than living in rural area (AOR = 2.37; 95% CI: 1.20, 4.67). In addition, women in Somali region were 87% less likely (AOR =0.13; 95% CI: 0.03, 0.52) to use modern contraceptive compared to women in Oromia region. Moreover, women who follow Muslim in religion were 51% less likely to use modern contraceptive (AOR =0.49; 95% CI: 0.36, 0.66) than Orthodox Christian followers (Table 4).

Measures of variation

According to the result in the null model, the ICC was 41.1% of the variations of modern contraceptive utilization among study subjects were attributed by difference at the cluster level, but the rest 58.9% were attributed to individual women factors. The final model indicates that the proportional change in variance (PCV) value of 0.606 indicates that both individual and community level factors were responsible for about 60.6% of the variation in modern contraceptive utilization between study subjects. When comparing models/fitness, deviance was used, this model was the best-fitting model, having the lowest deviance (3,551) (Table 4).

Table 4. Multivariable analyses for factors affecting modern contraceptive utilization (n=3822).

**Variables**	Model 0	Model 1 AOR (95% CI)	Model 2 AOR (95%CI)	Model 3 AOR (95%CI)
**Individual level Characteristics**				
**Age of respondents**				
15-24		1		1
25-34		1.39 (1.08, 1.77)		**1.29 (1.01, 1.66)***
35-49		0.58 (0.43, 0 .79)		**1.71 (1.37, 2.70)***

For peer review only - http://bmjopen.bmj.com/site/about/guidelines.xhtml

**Educational status of the respondents**			
No formal education	1		1
Primary education	1.15 (0.93, 1.44)		1.06 (0.85, 1.33)
Secondary and higher	1.33(0.78, 2.30)		1.02 (.59, 1.79)
**Husband education**			
No formal education	1		1
Primary education	1.14 (0.93,1.5)		1.05 (0.85, 1.29)
Secondary and higher	1.07 (0.76, 1.47)		0.96 (0.68, 1.34)
**Husband Occupation**			
Not working	1		1
Working	1.55 (1.16, 2.06)		**1.49 (1.03, 1.99)***
**Wealth index**			
Poor	1		1
Middle	1.24 (0.96, 1.59)		1.02 (0.79, 1.31)
Rich	1.53 (1.19, 1.98)		1.19 (0.92, 1.54)
**Media exposure**			
No	1		1
Yes	1.24 (0.99, 1.54)		1.15 (0.92, 1.43)
**Number of living children**			
None	1		1
1-4	2.18 (1.46, 3.25)		**2.20 (1.47, 3.30)***
5-8	1.70 (1.08, 2.69)		**1.74 (1.09, 2.77)***
≥ 9	1.10 (0.51, 2.39)		1.21 (0.55, 2.65)
**Visit of health facility in the last 12 months**			
No	1		1
Yes	1.32 (1.10, 1.58)		1.29 (0.74, 1.54)
**Partner's desire for children**			
Both want the same	1		
Husband wants more	0.75 (0.61, 0.94)		**0.77 (0.61, 0 .96)***
Husband wants fewer	0.84 (0.58, 1.22)		0.82 (0.56, 1.18)
Don't know	0.72 (0.57, 0 .90)		0.71 (0.56, 1.89)
**Community level variables**			
**Residency**			
Rural		1	1
Urban		2.86 (1.48, 5.55)	**2.37 (1.20, 4.67)***
**Community level media exposure**			
Low		1	1
High		1.92 (1.09, 3.39)	**1.77 (1.01, 3.08)***

For peer review only - http://bmjopen.bmj.com/site/about/guidelines.xhtml

**Community level poverty**				
High			0.32 (0.15, 0.69	0.33 (0.15, 1.70)
Low			1	**1**
**Community level education**				
Low			1	1
High			0.81(0.45, 1.43)	0.75 (0.43, 1.31)
**Region**				
Oromo			1	1
Afar			2.25 (0.87, 5.82)	2.19 (0.84, 5.7**)**
Somali			0.11 (0.03, 0.44)	**0.13 (.033, 0.52)***
**Religion**				
Orthodox Christian			1	1
Protestant			1.11 (0.83, 1.50)	1.08 (0.79, 1.47)
Muslim			0.52 (0.38, 0 .71)	**0.49 (0.36, 0.66)***
Others			0.61(0.33, 1.15)	0.61 (0.32, 1.16)
**Random effect results**				
Variance (%)	43.7	32.9	17.8	17.2
ICC (%)	41.1	33.3	20.6	19.5
MOR	17.1	14.8	10.9	10.7
PCV	Ref	24.7	59.3	60.6
**Model comparison**				
Deviance(-2LLR)	3,893	3,747	3,735	3,551

226 * = P-value < 0.05, ** = P value < 0.01, *** = P value < 0.001 ICC = Intra class correlation

227 coefficient, MOR =Median odds ratio, PCV = proportional change in variance. AOR=adjusted

228 odds ratio; CI= confidence interval.

For peer review only

Discussion

The aim of this study was to investigate the magnitude and associated factors of modern contraceptive utilization among reproductive age women in high fertility regions of Ethiopia. Slightly above one forth 29.75 % (95% CI: 28.2, 31.2) of participants were currently using modern contraceptive. This study is in line with study conducted in Dembia District.[43] The finding was lower than that reported from 2016 EDHS 35%[44], study done in Adama Town 47% and Ethiopia 51.6%.[45,46] However, this finding is higher than the previous studies conducted in Bale[22] ,Gumuz[47], Rural women in Ethiopia.[48] The difference might be due to the different design and sample size.

In this study, the usage of contraceptives was strongly related with women of reproductive age, specifically those age 25-34 and >35 were more likely to use contraceptives than those age 15-24. The findings were similar to those obtained by Nepal.[27] This may be because younger women (15-24 years old) perceive pregnancy risks are low.

The use of contraception by a wife is determined by her husband, and the wife is more likely to use contraception if her husband approves.[49] In this study, women have a higher likelihood of utilizing contraception if their husband holds a professional, technical, or managerial position. This husband's occupation may reflect husband's educational status and contributed for contraceptive use by women as indicated.[50] There is a higher possibility that these women can reside in urban regions.

The use of contraceptive methods was independently associated with the number of children. In comparison to a woman who had no children, women who had 1-4 children and women who had 5-8 children were nearly 2.2 and nearly 2 times more likely, respectively, to have used a modern form of contraception. Additionally, prior studies have shown that using modern contraceptives

For peer review only - http://bmjopen.bmj.com/site/about/guidelines.xhtml

is more likely when there are a lot of living children.[34,51-55] This might be because women without children might need to have children in order to have the ideal number of children.[56]

The current study also showed that the husband's desire to have more children was inversely related to modern contraceptive use. Women with husbands who want many children were 23% less likely to use modern contraceptives than women who want the same number of children with their husbands. This is congruent with the results of secondary data analyses of the Demographic and Health Survey of Bangladesh, Burkina Faso, and Mali which showed that a husband's desire to have children influenced a woman's use of modern contraception when she was of reproductive age.[57,58]

In regard to community level factors, according to this study, residence was independently associated with modern contraceptive use. Urban women of reproductive age are about three times more likely than rural women to use contemporary contraceptives. This finding is in line with secondary data analysis of Indian, Afghan, Nigerian, and Bangladeshi Demographic and Health Surveys, which showed that urban resident women were more likely than rural resident women to use contemporary contraceptives.[48,59-61] This could be caused by a variety of factors. Urban women tend to be more educated, earn more money, have better access to health facilities, and have better media access than rural women, all of which lead to higher modern contraceptive utilization rates. Women in rural areas also require more children to help them with field work, which negatively impacts their use of modern contraceptives.[40,62-66]

Women who were exposed to the community media had a higher likelihood of using modern methods of contraception than women who weren't. The finding is in line with the results .[34,67] [48,52,54] This is due to the possibility that exposure to mass media could play a significant role in raising awareness and inspiring women to take contemporary contraceptives.

For peer review only - http://bmjopen.bmj.com/site/about/guidelines.xhtml

In addition woman living in Somali region of Ethiopia was 87% less likely to use modern contraceptive compared to women in Oromia region. The reason could be different access to health information and different availability of maternal health services like family planning.[68] Moreover, women who were Muslim religious followers were less likely to use modern contraceptive than Orthodox Christian religious followers. It is possible that the low rate of family planning methods, such as the usage of contemporary contraceptives, among Muslims and Protestants in Ethiopia is the cause of this.[69] In addition, there is evidence that Muslims are less likely to approve of the use of contraceptives and have a negative attitude about family planning.[70]

Strengths and limitations

This study used most recent nationally representative data, which were collected with validated and standardized data collection tools. This also employed multilevel analysis (advanced model) that accounts the correlated nature of EDHS data in the determination of the estimate. Despite the above advantages, the cross sectional nature of the study does not show the cause and effect relationship between the outcome and the independent factors. Moreover, due to EDHS were secondary data, essential factors like attitude and knowledge about contraceptives, partner's perspectives on contraceptives, and fear of side effects were not available in the EDHS.

Conclusion

Modern contraceptives utilization in high fertility regions of Ethiopia was low. Women age, husband occupation, number of living children, husband's desired number of children, residency, community media exposure, region, and religion were significantly associated with modern contraceptive utilization. Therefore, to improve the utilization of modern contraceptives, public

For peer review only - http://bmjopen.bmj.com/site/about/guidelines.xhtml

297 health policymakers should consider creating awareness through mass media, male involvement
298 in family planning, as well as family planning programs, should be encouraged in rural areas.

299 **List of abbreviations**

300 AOR: Adjusted Odds Ratio; CI: Confidence interval; EDHS: Ethiopian Demographic and Health
301 Survey; ICC: Intra-class Correlation Coefficient; MOR: Median Odds Ratio; PCV: Proportional
302 Change in Variance

303 **Consent for publication**

304 Not applicable

305 **Availability of data and materials**

306 Data can be obtained upon reasonable request. The data used in this study will be made available
307 to the corresponding author upon reasonable request.

308 **Competing interests**

309 The authors declare that they have no competing interests.

310 **Funding**

311 Not applicable

312 **Authors' contributions**

313 TBB conceived the idea, extract the data, data analysis, and draft the manuscript. WDN, DAB,
314 and DBA participate in the data analysis, interpretation, and revising of the manuscript. All
315 authors have read and approved the final manuscript.

316 **Acknowledgments**

317 We are grateful to the EDHS programs, for the permission to use all the relevant EDHS data for
318 this study.

For peer review only - http://bmjopen.bmj.com/site/about/guidelines.xhtml

For peer review only

Ethical approval and consent to participate

Not applicable/No human participants included. Consent to participants is not applicable since the data is secondary and is available in the public domain. All the methods were conducted according to the Helsinki declarations. More details regarding DHS data and ethical standards are available online at (http://www.dhsprogram.com).

For peer review only

For peer review only - http://bmjopen.bmj.com/site/about/guidelines.xhtml

References

1. Cleland J, Bernstein S, Ezeh A, Faundes A, Glasier A, Innis J. Family planning: the unfinished agenda. *The lancet.* 2006;368(9549):1810-1827.
2. Kesetebirhan A. National guideline for family planning services in Ethiopia. *Federal Democratic Republic of Ethiopia, Ministry of Health.* 2011.
3. Nalwadda G, Namutebi M, Volgsten H. Health care providers' perceptions of family planning and contraception education for adolescents in Kampala, Uganda–A qualitative study. *Sexual & Reproductive Healthcare.* 2019;21:15-20.
4. Organization WH. Family planning: A global handbook for providers. 2018 Edition. *World Health Organization.* 2018.
5. Ajong AB, Njotang PN, Kenfack B, et al. Contraceptive method mix and preference: A focus on long acting reversible contraception in urban Cameroon. *PloS one.* 2018;13(8):e0202967.
6. Ugaz JI, Chatterji M, Gribble JN, Mitchell S. Regional trends in the use of short-acting and long-acting contraception accessed through the private and public sectors. *International Journal of Gynecology & Obstetrics.* 2015;130:E3-E7.
7. Tibaijuka L, Odongo R, Welikhe E, et al. Factors influencing use of long-acting versus short-acting contraceptive methods among reproductive-age women in a resource-limited setting. *BMC women's health.* 2017;17(1):1-13.
8. Hopkins K, Hubert C, Coleman-Minahan K, et al. Unmet demand for short-acting hormonal and long-acting reversible contraception among community college students in Texas. *Journal of American College Health.* 2018;66(5):360-368.
9. Espey E, Ogburn T. Long-acting reversible contraceptives: intrauterine devices and the contraceptive implant. *Obstetrics & Gynecology.* 2011;117(3):705-719.
10. Negash WD, Belachew TB, Asmamaw DB. Long acting reversible contraceptive utilization and its associated factors among modern contraceptive users in high fertility sub-Saharan Africa countries: a multi-level analysis of recent demographic and health surveys. *Archives of Public Health.* 2022;80(1):1-8.
11. Bahamondes L, Fernandes A, Monteiro I, Bahamondes MV. Long-acting reversible contraceptive (LARCs) methods. *Best Practice & Research Clinical Obstetrics & Gynaecology.* 2020;66:28-40.
12. Alemayehu M, Belachew T, Tilahun T. Factors associated with utilization of long acting and permanent contraceptive methods among married women of reproductive age in Mekelle town, Tigray region, north Ethiopia. *BMC pregnancy and childbirth.* 2012;12(1):1-9.
13. Getahun DS, Wolde HF, Muchie KF, Yeshita HY. Utilization and determinants of long term and permanent contraceptive methods among married reproductive age women at Janamora district, northwest Ethiopia. *BMC research notes.* 2018;11(1):1-6.
14. Zenebe CB, Adefris M, Yenit MK, Gelaw YA. Factors associated with utilization of long-acting and permanent contraceptive methods among women who have decided not to have more children in Gondar city. *BMC women's health.* 2017;17(1):1-7.
15. Creanga AA, Gillespie D, Karklins S, Tsui AO. Low use of contraception among poor women in Africa: an equity issue. *Bulletin of the world Health Organization.* 2011;89:258-266.
16. Miller K, Miller RA, Askew I, Horn MC, Ndhlovu L. Clinic-based family planning and reproductive health services in Africa: findings from situation analysis studies. 1998.
17. Alenoghena I, Yerumoh S, Momoh A. Knowledge, attitude and uptake of family planning services among women of reproductive age group attending outpatient clinic at a tertiary health institution in Edo State, Nigeria. *Journal of Public Health and Epidemiology.* 2019;11(3):63-70.

For peer review only - http://bmjopen.bmj.com/site/about/guidelines.xhtml

18. Sharma SK, Pratap N, Ghimire DR. Ethnic differentials of the impact of Family Planning Program on contraceptive use in Nepal. *Demographic Research.* 2011;25:837-868.
19. Kibria G, Hossen S, Barsha RAA, Sharmeen A, Paul SK, Uddin SI. Factors affecting contraceptive use among married women of reproductive age in Bangladesh. *Journal of Molecular Studies and Medicine Research.* 2016;2(01):70-79.
20. Peer N, London L, Morojele N. Factors associated with contraceptive use in a rural area in Western Cape Province. *South African Medical Journal.* 2013;103(6):406-412.
21. Kisia Malalu P, Koskei A, Too R, Chirchir A. Determinants of use of modern family planning methods: A case of Baringo North District, Kenya. 2014.
22. Belda SS, Haile MT, Melku AT, Tololu AK. Modern contraceptive utilization and associated factors among married pastoralist women in Bale eco-region, Bale Zone, South East Ethiopia. *BMC health services research.* 2017;17(1):1-12.
23. Mekonnen W, Worku A. Determinants of low family planning use and high unmet need in Butajira District, South Central Ethiopia. *Reproductive health.* 2011;8(1):1-8.
24. Lakew Y, Reda AA, Tamene H, Benedict S, Deribe K. Geographical variation and factors influencing modern contraceptive use among married women in Ethiopia: evidence from a national population based survey. *Reproductive health.* 2013;10(1):1-10.
25. Tilahun T, Coene G, Luchters S, et al. Family planning knowledge, attitude and practice among married couples in Jimma Zone, Ethiopia. *PloS one.* 2013;8(4):e61335.
26. Adebowale SA, Palamuleni ME. Determinants of unmet need for modern contraception and reasons for non-use among married women in rural areas of Burkina Faso. *African Population Studies.* 2014:499-514.
27. Fotso JC, Izugbara C, Saliku T, Ochako R. Unintended pregnancy and subsequent use of modern contraceptive among slum and non-slum women in Nairobi, Kenya. *BMC pregnancy and childbirth.* 2014;14(1):1-10.
28. Apanga PA, Adam MA. Factors influencing the uptake of family planning services in the Talensi District, Ghana. *Pan African Medical Journal.* 2015;20(1).
29. Medhanyie AA, Desta A, Alemayehu M, et al. Factors associated with contraceptive use in Tigray, North Ethiopia. *Reproductive health.* 2017;14(1):1-11.
30. Mohammed A, Woldeyohannes D, Feleke A, Megabiaw B. Determinants of modern contraceptive utilization among married women of reproductive age group in North Shoa Zone, Amhara Region, Ethiopia. *Reproductive health.* 2014;11(1):1-7.
31. Kahsay ZH, Hiluf MK, Shamie R, Tadesse Y, Bazzano AN. Pregnant Women's intentions to deliver at a health Facility in the Pastoralist Communities of Afar, Ethiopia: an application of the health belief model. *International Journal of Environmental Research and Public Health.* 2019;16(5):888.
32. Sedlander E, Bingenheimer JB, Edberg MC, Rimal RN, Shaikh H, Munar W. Understanding modern contraception uptake in one Ethiopian community: a case study. *Reproductive health.* 2018;15(1):1-8.
33. Fantay Gebru K, Mekonnen Haileselassie W, Haftom Temesgen A, Oumer Seid A, Afework Mulugeta B. Determinants of stunting among under-five children in Ethiopia: a multilevel mixed-effects analysis of 2016 Ethiopian demographic and health survey data. *BMC pediatrics.* 2019;19(1):1-13.
34. Abate MG, Tareke AA. Individual and community level associates of contraceptive use in Ethiopia: a multilevel mixed effects analysis. *Archives of Public Health.* 2019;77(1):1-12.
35. African countries with the highest fertility rate | Statista https://worldpopulationreview.com/countries/total-fertility-rate. cited on December 8, 2021.

For peer review only - http://bmjopen.bmj.com/site/about/guidelines.xhtml

36. Csa I. Central statistical agency (CSA)[Ethiopia] and ICF. *Ethiopia demographic and health survey, Addis Ababa, Ethiopia and Calverton, Maryland, USA.* 2016.
37. Corsi DJ, Neuman M, Finlay JE, Subramanian S. Demographic and health surveys: a profile. *International journal of epidemiology.* 2012;41(6):1602-1613.
38. Croft T, Marshall A, Allen C. Guide to DHS statistics. Rockville: ICF; 20182018.
39. Eshete T, Kumera G, Bazezew Y, Mihretie A, Marie T. Determinants of inadequate minimum dietary diversity among children aged 6–23 months in Ethiopia: secondary data analysis from Ethiopian Demographic and Health Survey 2016. *Agriculture & Food Security.* 2018;7(1):1-8.
40. Gebre MN, Edossa ZK. Modern contraceptive utilization and associated factors among reproductive-age women in Ethiopia: evidence from 2016 Ethiopia demographic and health survey. *BMC Women's Health.* 2020;20(1):1-14.
41. Liyew AM, Teshale AB. Individual and community level factors associated with anemia among lactating mothers in Ethiopia using data from Ethiopian demographic and health survey, 2016; a multilevel analysis. *BMC Public Health.* 2020;20(1):1-11.
42. Getaneh T, Negesse A, Dessie G, Desta M, Moltot T. Predictors of unmet need for family planning in Ethiopia 2019: a systematic review and meta analysis. *Archives of Public Health.* 2020;78(1):1-11.
43. Debebe S, Limenih MA, Biadgo B. Modern contraceptive methods utilization and associated factors among reproductive aged women in rural Dembia District, northwest Ethiopia: Community based cross-sectional study. *International Journal of Reproductive BioMedicine.* 2017;15(6):367.
44. Demographic CE. Health Survey-2011. Central Statistical Agency Addis Ababa. Ethiopia ICF International Calverton, Maryland, USA. 20122016.
45. Biruk T, Assefa H, Georges R. The prevalence of Covert use of contraceptives in Nazareth/Adama town. *Eur J Contracept Reprod Health Care.* 2008;13(2):63.
46. Abraha MW, Nigatu TH. Modeling trends of health and health related indicators in Ethiopia (1995-2008): a time-series study. *Health Research Policy and Systems.* 2009;7(1):1-17.
47. Adane AA, Bekele YA, Melese E, Worku GT, Netsere HB. Modern contraceptive utilization and associated factors among married gumuz women in Metekel Zone North West Ethiopia. *BioMed Research International.* 2020;2020.
48. Fenta SM, Gebremichael SG. Predictors of modern contraceptive usage among sexually active rural women in Ethiopia: A multi-level analysis. *Archives of Public Health.* 2021;79(1):1-10.
49. Kamal N. The influence of husbands on contraceptive use by Bangladeshi women. *Health policy and planning.* 2000;15(1):43-51.
50. Gubhaju B. The influence of wives' and husbands' education levels on contraceptive method choice in Nepal, 1996-2006. *International Perspectives on Sexual and Reproductive Health.* 2009:176-185.
51. Idris H. Factors Affecting the Use of Contraceptive in Indonesia: Analysis from the National Socioeconomic Survey (Susenas). *KEMAS: Jurnal Kesehatan Masyarakat.* 2019;15(1):117-123.
52. Rutaremwa G, Kabagenyi A, Wandera SO, Jhamba T, Akiror E, Nviiri HL. Predictors of modern contraceptive use during the postpartum period among women in Uganda: a population-based cross sectional study. *BMC public health.* 2015;15(1):1-9.
53. Musa A, Assefa N, Weldegebreal F, Mitiku H, Teklemariam Z. Factor associated with experience of modern contraceptive use before pregnancy among women who gave birth in Kersa HDSS, Ethiopia. *BMC public health.* 2016;16(1):1-6.

For peer review only

54. Nsanya MK, Atchison CJ, Bottomley C, Doyle AM, Kapiga SH. Modern contraceptive use among sexually active women aged 15–19 years in North-Western Tanzania: results from the Adolescent 360 (A360) baseline survey. *BMJ open.* 2019;9(8):e030485.

55. Devita VD, Rosliza A, Suriani I. Prevalence of modern contraceptive use among reproductive women with hypertension and diabetes in a government hospital in BATAM, Indonesia and its socio-demographic determinants. *International Journal of Public Health and Clinical Sciences.* 2018;5(4):297-278.

56. Matovu JK, Makumbi F, Wanyenze RK, Serwadda D. Determinants of fertility desire among married or cohabiting individuals in Rakai, Uganda: a cross-sectional study. *Reproductive health.* 2017;14(1):1-11.

57. O'Regan A, Thompson G. Indicators of young women's modern contraceptive use in Burkina Faso and Mali from Demographic and Health Survey data. *Contraception and reproductive medicine.* 2017;2(1):1-8.

58. Hossain M, Khan M, Ababneh F, Shaw J. Identifying factors influencing contraceptive use in Bangladesh: evidence from BDHS 2014 data. *BMC public health.* 2018;18(1):1-14.

59. Ezeh OK, Agho KE, Dibley MJ, Hall J, Page AN. The impact of water and sanitation on childhood mortality in Nigeria: evidence from demographic and health surveys, 2003–2013. *International journal of environmental research and public health.* 2014;11(9):9256-9272.

60. Haq I, Sakib S, Talukder A. Sociodemographic factors on contraceptive use among ever-married women of reproductive age: evidence from three demographic and health surveys in Bangladesh. *Medical Sciences.* 2017;5(4):31.

61. Dey AK. Socio-demographic determinants and modern family planning usage pattern-an analysis of National Family Health Survey-IV data. *Int J Community Med Public Heal.* 2019;6(2):738.

62. Tekelab T, Melka AS, Wirtu D. Predictors of modern contraceptive methods use among married women of reproductive age groups in Western Ethiopia: a community based cross-sectional study. *BMC women's health.* 2015;15(1):1-8.

63. Johnson OE. Determinants of modern contraceptive uptake among Nigerian women: evidence from the national demographic and health survey. *African journal of reproductive health.* 2017;21(3):89-95.

64. Osmani AK, Reyer JA, Osmani AR, Hamajima N. Factors influencing contraceptive use among women in Afghanistan: secondary analysis of Afghanistan Health Survey 2012. *Nagoya journal of medical science.* 2015;77(4):551.

65. Adebowale AS, Gbadebo B, Afolabi FR. Wealth index, empowerment and modern contraceptive use among married women in Nigeria: are they interrelated? *Journal of Public Health.* 2016;24(5):415-426.

66. Adebowale SA, Adedini SA, Ibisomi LD, Palamuleni ME. Differential effect of wealth quintile on modern contraceptive use and fertility: evidence from Malawian women. *BMC women's health.* 2014;14(1):1-13.

67. Agbadi P, Tagoe E, Akosua AF, Owusu S. *A multilevel analysis of predictors of modern contraceptive use among reproductive age women in Sierra Leone: insight from demographic and Health surveys*: Center for Open Science;2019.

68. Ababa A. Ethiopia. Abstract available from: https://wfpha. confex. com/wfpha/2012/webprogram. *Paper10587. html [Google Scholar].* 2013.

69. Roudi-Fahimi F. *Islam and family planning*: Population Reference Bureau Washington, DC; 2004.

70. Duze MC, Mohammed IZ. Male knowledge, attitude, and family planning practices in Northern Nigeria. *African Journal of Reproductive Health.* 2006;10(3):53-65.

For peer review only - http://bmjopen.bmj.com/site/about/guidelines.xhtml

508 **List of Legends**

509 Figure 1. Schematic illustration of women included in high fertility regions of Ethiopia

510 Figure 2. Magnitude of modern contraceptive utilization in high fertility regions of Ethiopia

For peer review only

For peer review only - http://bmjopen.bmj.com/site/about/guidelines.xhtml

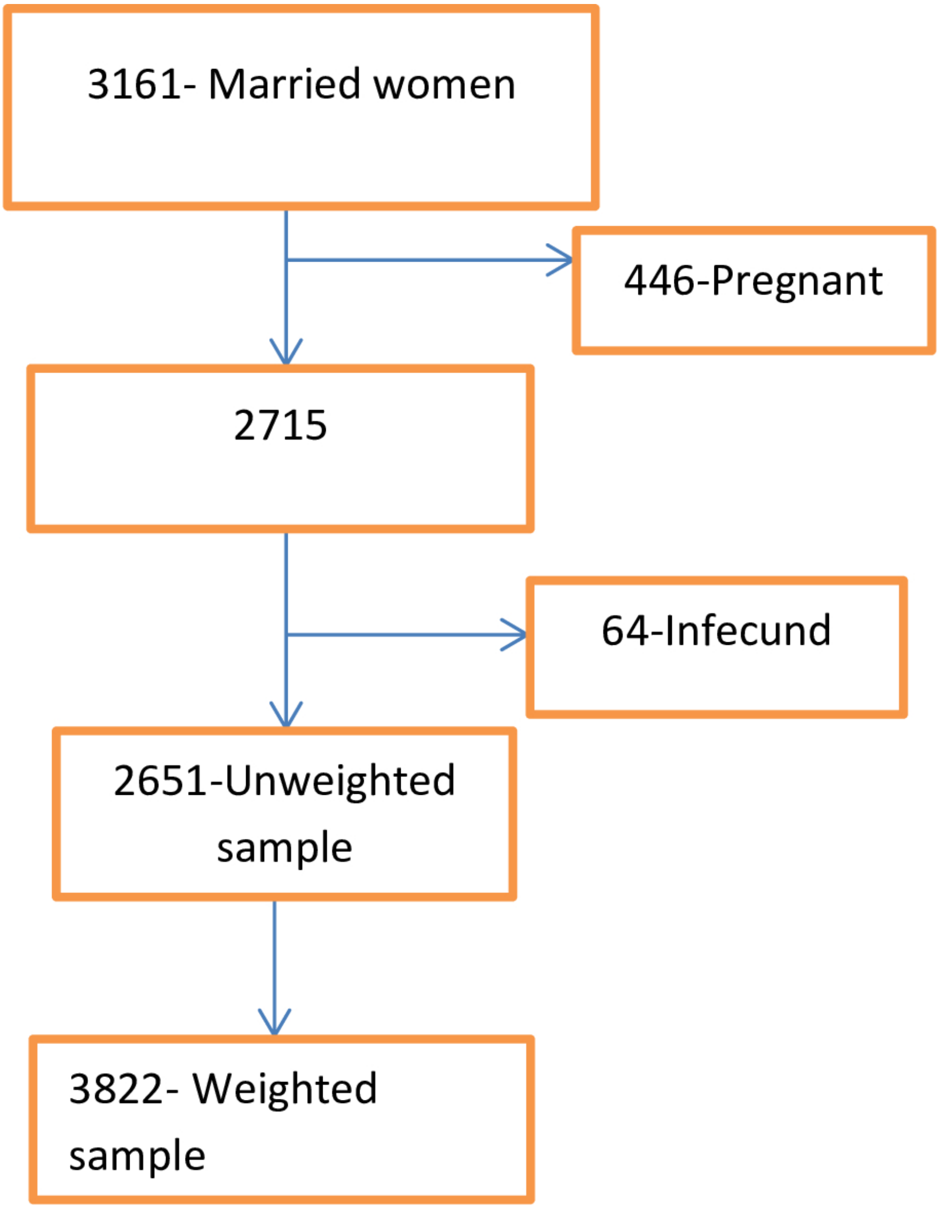

Figure 1. Schematic illustration of women included in high fertility regions of Ethiopia

77x98mm (300 x 300 DPI)

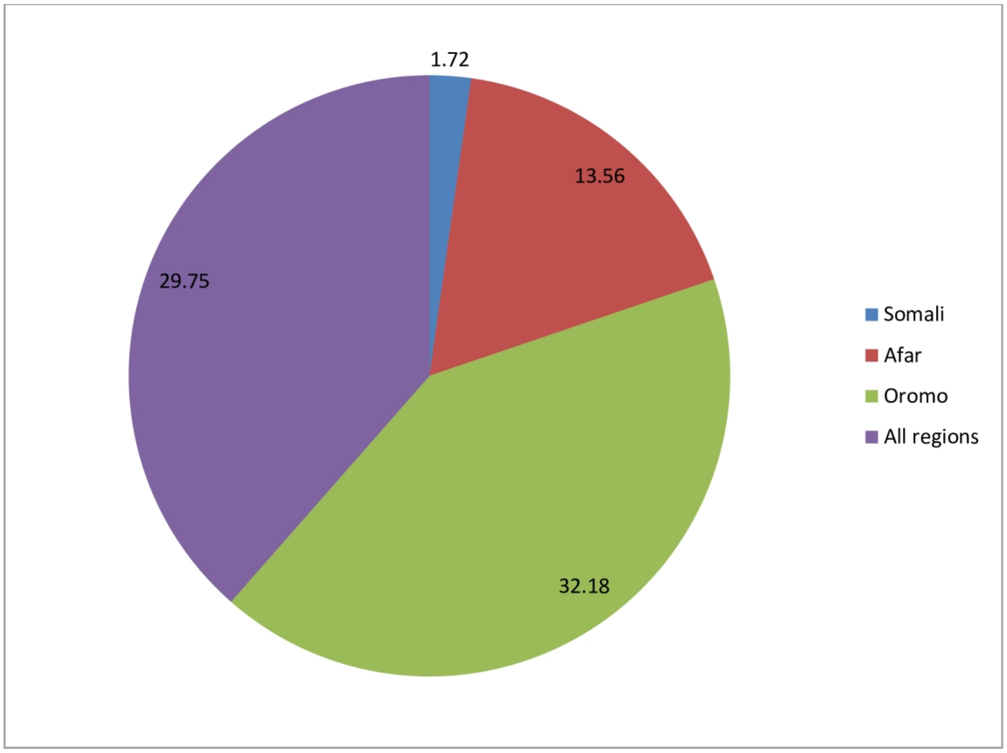

Figure 2. Magnitude of modern contraceptive utilization in high fertility regions of Ethiopia

156x116mm (300 x 300 DPI)

STROBE Statement—Checklist of items that should be included in reports of ***cross-sectional studies***

**Items**	**Number**	**Recommendations**
**Title and abstract**	1	(*a*) Indicate the study's design with a commonly used term in the title or the abstract **page 2, line number 24**
		(b) provide in the abstract an informative and balanced summary of what was done and what was found **Page 2, line number 27-39**
**Introduction**		
Background/rational	2	Explain the scientific background and rationale for the investigation being reported **Page 4-5, line number 59-100**
Objective	3	State specific objectives, including any prespecified hypotheses **Page 2, line number 22-23**
**Methods**		
Study design	4	Present key elements of study design early in the paper **page 6, line number 103-104**
Setting	5	Describer the setting, location, and relevant dates including period of recruitment and data collection **Page number 6, line number 108-111**

For peer review only

Participants	6	Give the eligibility criteria, and the sources and methods of selection of participants<br>**Page 2, line number 26**
		Clearly define all outcomes, exposures, predictors, potential confounders, and effect modifier, Give diagnostic, if applicable<br>**Page 7-8 line number 131-147**
Data source and measurement	7	**Page 6-7 line number 116-128**
Bias	8	Describe any efforts to address potential sources of bias<br>**N/A**
Study size	9	Explain how the study design was arrived<br>**Page 6 , line number 105-108**
Quantitative variables	10	Explain how quantitative variables were handled in the analysis , if applicable describe which groupings chose and why<br>**Page7 -8, line number 131-147**
Statistical methods	11	(a)Describe all statistical methods, including those used to control for confounding<br>**Page 8, line number 148-159**

For peer review only

		(*b*) Describe any methods used to examine subgroups and interactions<br>**NA**
		(*c*) Explain how missing data were addressed<br>**NA**
		(*d*) If applicable, describe analytical methods taking account of sampling strategy<br>**NA**
		(*e*) Describe any sensitivity analyses<br>**NA**
**Results**		
Descriptive data	12	Give characteristics of study participants (eg, demographic, clinical, social) and information on exposure and potential confounders<br>**Page 9, line number 171-181**
Outcome data	13	Report numbers of outcome events or summary measures<br>**Page 10, line number 183-184**
Main results	14	(a) Give unadjusted estimates and, if applicable, confounder adjusted estimates and their precision (e.g., 95% confidence interval).Make clear which confounders were adjusted for and why they were included<br>**Page 9-10, line number 187-207**

For peer review only

		(b) If relevant, consider translating estimates of relative risk into absolute risk for a meaningful time period<br>**NA**
Other analysis	15	Report other analyses doing analyses of subgroups and interactions, and sensitive analysis<br>**NA**
**Discussion**		
Key result	16	Summaries key results with reference to study objectives<br>**Page 12, line number 119-224**
Limitations	17	Discuss limitations of the study, taking into account sources of potential bias or imprecision. discuss both direction and magnitude of any bias<br>**Page 14 , page 274-278**
Interpretation	18	Give a cautious overall interpretation of results considering objectives, limitations, multiplicity of analysis, result from similar studies, and other relevant evidence<br>**Page 12-14, line number 217-278**
Generalizability	19	Discuss the generalizability (external validity) of the study results<br>**Page14-15, line number 280-285**

For peer review only

Funding	20	Give the source of funding and the role of the funders for the present study and if applicable for the original study on which the present article is based<br>**Page 15, line number 298**

Note: An explanation and elaboration article discusses each checklist item and gives methodological background published examples of transport reporting. The STROBE checklist is best used in conjunction with this articles (freely available on the web sites of PloS Medicine at http://www.plosmedicene.org/.Annals of internal medicine http://www.annals.org/, and Epidemiology at http://www.epidem.com/ information on the STROBE initiative is available at www.strobe-statement.org.

For peer review only
